# Nanotoxicity of Porous Silica Nanoparticles: Physicochemical Properties and Mechanistic Cellular Endpoints

**DOI:** 10.3390/nano15231766

**Published:** 2025-11-25

**Authors:** Trisha Patel, Callum Clipstone, Umakhanth Venkatraman Girija, Zeeshan Ahmad, Neenu Singh

**Affiliations:** 1 Leicester School of Allied Health Sciences, De Montfort University, Leicester LE1 9BH, UK; trishapatel031@gmail.com (T.P.); p2601417@my365.dmu.ac.uk (C.C.); umakhanth.venkatramangirija@dmu.ac.uk (U.V.G.); 2 Division of Pharmacy and Optometry, School of Health Sciences, University of Manchester, Manchester M13 9PL, UK; zeeshan.ahmad@manchester.ac.uk; 3 Leicester School of Pharmacy, De Montfort University, Leicester LE1 9BH, UK

**Keywords:** nanotoxicity, porous silica nanoparticles, cytotoxicity, genotoxicity, immunogenicity, physicochemical characterization, clinical translation, safety

## Abstract

This review provides a comprehensive overview of the fundamental aspects of nanoparticles (NPs), emphasizing their physicochemical properties and biological interactions, with particular focus on porous silica nanoparticles (PSNs). The review provides information on the Safe-by-design (SbD) S.A.F.E. (Standardised characterization, Assessment of biocompatibility, Facilitation of toxicity and exposure routes and Evaluation of clinical translation) framework. It discusses critical factors influencing NP toxicity and cellular uptake, including particle size, shape, pore size, surface charge, surface functionalisation, and crystallinity. The review also examines exposure routes of NPs—inhalation, dermal, oral, systemic and mucosal—and their subsequent biological effects. A key section is dedicated to the formation of the protein corona, a critical determinant of NP fate in biological systems, and its influence on circulation time, immune clearance and cellular responses. Particular attention is given to assessing the biological interactions of the PSNs and the mechanisms underlying PSN-induced cytotoxicity and genotoxicity, with a focus on the assays commonly employed to evaluate these effects. The review explores the use of gene expression profiling as a powerful tool to elucidate the molecular mechanisms underlying nanoparticle-induced cellular changes. This review aims to provide an integrated perspective on the SbD considerations and safety implications of nanomaterials. It highlights the need for a deeper understanding of complex biological interactions to establish SbD principles and enable the translation of PSNs into clinical applications. Finally, current regulatory frameworks and guidelines for testing nanomaterials, including PSNs, that support their safe and sustainable development are discussed.

## 1. Introduction

**Figure 1:**
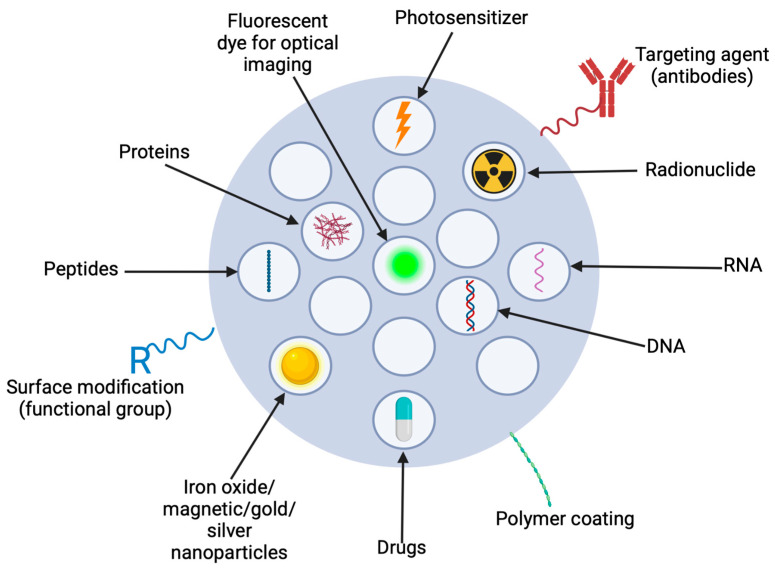
Functionalisation and loading of PSNs for biomedical applications (Created in BioRender. Patel, T. (2025) https://BioRender.com/7bksooi). This schematic diagram illustrates the diverse functionalisation and cargo-loading capabilities of PSNs. PSNs can be engineered to incorporate various molecules, enhancing their functionality for targeted drug delivery, imaging, and therapeutic applications.

Porous silica nanoparticles (PSNs) have become widely used nanocarriers in biomedical applications, particularly drug delivery. Their high surface area, tunable pore size, and easy functionalisation make them ideal candidates as delivery vehicles for a variety of therapeutic agents, including small-molecule drugs, proteins, and nucleic acids [1,2]. These properties enable controlled drug delivery, thereby improving bioavailability and reducing systemic toxicity. Additionally, their stability and tunability in biological membrane interactions contribute to their versatility across various applications [3,4]. The diverse strategies for functionalizing and loading PSNs with therapeutic agents are summarized in Figure 1, highlighting their adaptability for targeted biomedical applications.

This review is novel in that it provides an integrated, multidisciplinary perspective on PSNs, combining physicochemical characterization, biological interactions and Safe-by-Design (SbD) principles. Previous reviews have focused on individual toxicological endpoints such as cytotoxicity or genotoxicity; however, this work encompasses a broader spectrum of biological responses, including protein corona formation and immunogenicity, and links them to design parameters and exposure pathways. Furthermore. SbD approaches have been applied generally to nanomaterials; however, no comprehensive SbD principles have focused on PSNs, making this review the first to evaluate their safety within the S.A.F.E. framework and clinical translation.

### 1.1. Applications and Clinical Translation of PSNs

The biomedical application of PSNs represents a significant advancement in nanomedicine, connecting innovative bench-scale designs with human safety, pharmacokinetics, and efficacy considerations. Silicon dioxide has been classified as “generally recognized as safe” (GRAS) by the U.S. FDA, enabling its widespread use in food additives and cosmetic products [5,6]. This safety recognition provides a strong foundation for its clinical translation, supporting the development of PSNs as platforms for drug delivery, diagnostic imaging and other therapeutic applications. While the translation into late-phase therapeutic trials is still in its early stages, numerous first-in-human and early clinical trials have shown that silica-based nanomaterials can be safely administered at tracer or microdosing levels, display favourable pharmacokinetics, particularly renal clearance for ultrasmall silica and effectively deliver functional payloads or imaging contrast to targeted sites [7]. Table 1 shows clinical trials that have used porous or mesoporous silica nanoparticles (MSNs). The primary translational challenge involves balancing multifunctionality, such as targeting ligands, stimuli-responsive release, imaging moieties and factors like manufacturability, reproducibility, biocompatibility, and predictable in vivo degradation and clearance [5,8].

PSNs can deliver chemotherapy by encapsulating drugs such as doxorubicin, paclitaxel, and irinotecan within their porous structure and releasing them via controlled or stimulus-responsive mechanisms, such as pH or enzymes, directly to tumour cells [16,17,18,19,20,21]. While PSNs have demonstrated significant potential in preclinical studies, their clinical translation remains limited. Several studies have explored their efficacy in chemotherapy applications, highlighting their potential to enhance drug delivery and reduce systemic toxicity. Another important application of PSNs is their use as carriers for protein and peptide therapeutics. Common challenges with protein and peptide therapeutics include enzymatic degradation, poor stability, and limited cellular uptake. The properties of PSNs enable efficient loading of peptides and proteins, protection from harsh biological environments and targeted release, making them a multifunctional carrier for protein and peptide therapeutics [22,23]. MSNs can protect proteins from degradation and maintain their biological activity, facilitating targeted intracellular delivery of rapidly degrading peptides and proteins [24,25,26]. RNA and gene delivery play a vital role in modern therapeutics, allowing modulation of gene expression, inhibition of pathogenic pathways and repair of genetic abnormalities [27]. However, there is a major challenge in effective delivery due to the instability of nucleic acids, their susceptibility to enzymatic degradation, and limited cellular uptake. To overcome this, PSNs offer a promising solution, as their modifiable properties enable efficient encapsulation, protection, and targeted release [28]. Numerous studies have demonstrated that PSNs can be functionalised or loaded with fluorescent probes to aid in cellular uptake studies and cancer imaging. Owing to their high surface area and tunable porosity, PSNs can minimize photobleaching and dye leakage, thereby enhancing targeted fluorescence imaging, demonstrating their potential for diagnostics and theragnostic applications [29,30]. Furthermore, PSNs have been utilized as carriers for bioimaging agents, improving fluorescence and MRI contrast, which could enhance early disease detection and precision diagnostics. Research showed that MSNs doped with gadolinium oxide significantly improved T1 MRI contrast compared with the conventional contrast agent, gadolinium diethylene triamine pentaacetate, likely due to the structure of the MSNs, which increased surface area [31]. Lastly, PSNs have been used in antimicrobial therapy. Their high surface area, easy functionalisation, and controllable loading and release kinetics make PSNs an excellent carrier for antibiotics, antimicrobial peptides and metal-based agents [32].

### 1.2. Double-Edged PSNs: Potential Toxicity Risks

Although PSNs have unique and promising characteristics, a major concern in their therapeutic use is potential toxicity to the human body. Various studies have described mechanisms by which nanoparticles (NPs) may induce toxic effects, including oxidative stress, genotoxicity, inflammation, extent of cellular uptake, and disruption of cellular functions, highlighting the importance of careful design and surface modifications to mitigate potential adverse effects [33,34,35].

Silica-based NPs are considered biocompatible, but their interactions with biological systems can vary depending on their physicochemical properties, such as particle size, surface charge, and functionalisation [36]. To mitigate toxicity while maximizing therapeutic efficacy, research has focused on modifying the surface properties of PSNs to enhance their biocompatibility. PEG functionalisation, zwitterionic coatings, and biomimetic surface modifications are among the strategies used to increase NP stability, extend circulation time, and reduce immune recognition [37,38,39]. Zwitterionic coatings use molecules that have both a positive and a negative charge to produce a neutral surface which repels proteins, while biomimetic surface modifications coat NPs with natural biological components, such as cell membranes or proteins, to ensure maximum biocompatibility and reduce toxicity [40,41,42,43].

To fabricate PSNs for safe and effective biomedical applications, a better understanding of the relationship between physicochemical properties and biological responses is essential. Therefore, comprehensive characterization and rigorous safety evaluations are crucial to ensuring their successful translation into clinical use.

## 2. Safe-by-Design: Modulating Nanoparticle Physicochemical Properties to Control and Minimize PSN Toxicity

### 2.1. Safe-by-Design Principles of PSNs

**Figure 2:**
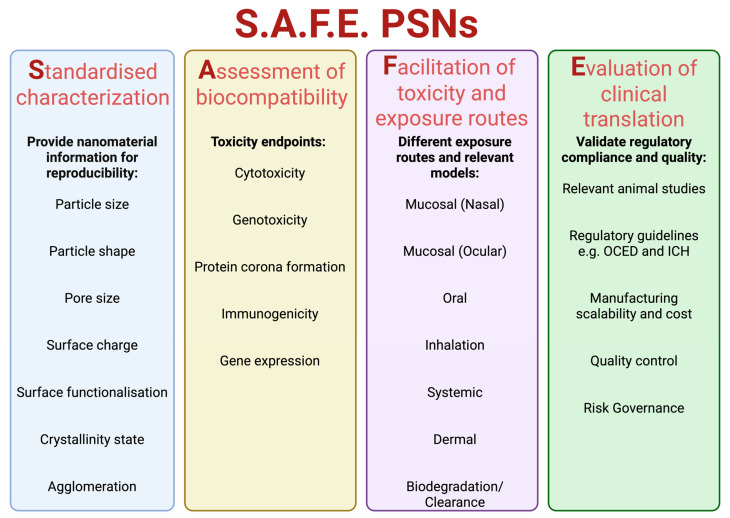
Safe-by-Design Principles of PSNs (Created in BioRender. Patel, T. (2025) https://BioRender.com/o2qwh8p). The S.A.F.E. framework is based on Standardised characterization, Assessment of biocompatibility, Facilitation of toxicity and exposure routes and Evaluation of clinical translation.

Nanoparticles are at the forefront of modern nanotechnology, from biomedical applications to cosmetic uses. As the market for nanoproducts grows, the importance of nano safety procedures to mitigate potential hazards to engineers and consumers becomes increasingly crucial. SbD strategies enable a more calculated and careful approach to the clinical translation of NPs [44,45]. This approach not only benefits the patient or consumer but also reduces harmful by-products that would otherwise harm the environment. In this section, various SbD strategies approaches for PSNs have been discussed, using the S.A.F.E. PSNs framework based on the GoNanoBioMat SbD approach [46]. The S.A.F.E. framework (Figure 2) discusses Standardised characterization, Assessment of biocompatibility, Frameworks for understanding nanoparticle toxicity, exposure routes, and Relevant Model and Evaluation of clinical translation.

PSNs have unique characteristics; their biological interactions and toxicity are primarily determined by their physicochemical properties, such as size, shape, surface charge, pore size, surface functionalisation, and crystallinity. Such features dictate the interfaces between PSNs and biological membranes, cellular uptake pathways, biodistribution, and the mechanisms of toxicity. Therefore, knowledge of these parameters is critical for engineering safer and more effective nanomedicine applications [47].

### 2.2. Standardised Characterization

A critical aspect of safe advanced nanomaterial design lies in standardizing the physicochemical attributes that influence PSN safety profiles. Key attributes that govern therapeutic delivery and ultimately biocompatibility, include particle size, particle shape, pore size, surface charge, surface functionalisation, and crystalline structure. They influence biological interactions, including protein corona formation, cellular uptake pathways, biodistribution, and clearance [5,48,49,50].

Alterations in nanomaterial features could result in acute and chronic biological effects [51]. Standardised characterization is also important to ensure reproducibility from laboratories to scale up manufacturing. The synthesis of NPs in preclinical studies usually involves template, sol–gel or hydrothermal methods, which are known to exhibit batch-to-batch variability [52]. This poses a significant risk that clinical study results may not be reliable. Therefore, to overcome this, standardized characterization is essential to produce safe and reliable PSNs.

#### 2.2.1. Particle Size

When engineering and producing PSNs, determining an optimal size is a significant aspect of SbD principles. As confirmed in several studies, smaller NPs (<100 nm) generally exhibit greater cellular uptake than larger particles, primarily due to their cellular uptake via endocytosis [53,54]. For example, a study found that amorphous silica nanoparticles (SiNPs) with an average diameter of 42 ± 3 nm could penetrate epidermal cells. However, particles greater than 75 nm could not penetrate human skin, even when mild barrier perturbation was induced [55]. While this enhanced permeability can be therapeutically advantageous, it also poses challenges, as small NPs (10–100 nm) are more likely to cross biological barriers, including the blood–brain barrier (BBB), suggesting that NPs could accumulate in the central nervous system and cause adverse side effects [56]. Furthermore, the renal system is the primary route of excretion for these NPs; kidney uptake may increase the risk of nephrotoxicity. Therefore, the design of PSNs must be optimized to minimize such risks by controlling particle size [57]. Particle size also influences the type of toxicity observed. Smaller particles (129.7 ± 2.5 nm) have been seen to cause cytotoxicity, while larger particles (355.4 ± 41.0 nm) can trigger increased secretion of interleukin-6 (IL-6) and tumour necrosis factor-alpha (TNF-α), and initiate inflammation [58,59]. In a comparative analysis, SiNPs of different sizes (10 nm and 100 nm) showed that the smallest particles induced the greatest cytotoxicity and oxidative stress, associated with their larger surface area-to-volume ratio, and increased reactive oxygen species (ROS) generation [60]. Similarly, research on RAW264.7 macrophages has shown that smaller amorphous silica NPs (70 nm) were more toxic than those of 300 and 1000 nm, suggesting that particle size is a factor in NP toxicity [61]. In contrast, larger NPs (>200 nm) predominantly underwent internalization by phagocytosis with slower clearance rates, leading to longer retention times in organs such as the liver and spleen [62]. These findings highlight the need for size optimization, balancing therapeutic efficiency and minimizing toxicity.

#### 2.2.2. Particle Shape

The shape of PSNs affects cellular uptake efficiency, biodistribution, and mechanical interactions with biological membranes. Although spherical NPs are most widely studied and used because of their uniform uptake and predictable behaviour, elongated or rod-shaped NPs may exhibit different biological interactions. For example, rod-shaped MSNs showed better cellular uptake in macrophages compared to corresponding spherical NPs [63,64]. The elongated shape facilitates alignment with the cell membrane, enhancing uptake via membrane wrapping mechanisms, and induces greater mechanical stress on cellular membranes, resulting in membrane rupture, oxidative stress and higher inflammatory responses [65]. Other studies have shown that nanorods and nanowires have long circulation half-lives in vivo, as their non-spherical shape reduces opsonisation and clearance (given their relatively high aspect ratio, making membrane wrapping suboptimal) by the mononuclear phagocyte system (MPS) [66,67,68,69,70]. This suggests that while specific shapes may increase toxicity in vitro, the in vivo behavior may differ, necessitating careful evaluation of both in vitro and in vivo toxicity models.

#### 2.2.3. Pore Size

Research has shown that larger pores (6–10 nm) enhance drug-loading capacity have accelerate release rates, and are useful for treatments [57,71]. While smaller pores (<5 nm) support prolonged drug release for the treatment of chronic diseases or long-term chemotherapy [72,73]. A study found that MSNs with 2.7 nm pores exhibited lower cytotoxicity compared to those with larger pore sizes of 7.39 nm, possibly due to reduced protein aggregation and minimal immune activation [57]. However, NPs with larger pores (>10 nm) increase surface area and reactivity, promoting interactions with cell components that generate ROS [74]. These findings highlight the importance of tuning pore size to optimize drug loading efficiency while minimizing toxicity.

#### 2.2.4. Surface Charge

The surface charge has been shown to determine their cellular uptake, including phagocytosis, underlying toxicity and subsequent clearance. It has been widely accepted that compared to anionic NPs, cationic NPs can promote greater internalization via electrostatic attraction to negatively charged cell membranes, increase cytotoxicity, induce an immune response and upregulate apoptotic pathways [75,76]. Additionally, serum proteins can modulate these interactions. For example, a study using glioblastoma stem cells indicated that positively charged MSNs functionalised with polyethyleneimine (PEI) (PEI-MSNs) were toxic because PEI-MSNs internalized into lysosomes, rupturing lysosomal membranes [77]. Also, in another study of AgNPs and human epidermal keratinocytes (HEK), serum proteins (albumin and IgG) were found to reduce AgNP uptake of 110 nm citrate-coated AgNPs [78]. These studies collectively highlight the importance of surface charge in NP-cell interactions.

Surface modification of MSN with PEI-PEG to impart a positive charge has been proven to be one of the most effective methods to improve nanoparticle uptake and biodistribution, reduce opsonisation, and endow antitumor properties [79].

However, the decrease in opsonisation and phagocytosis allows for a longer exposure time. This potentially increases the inflammatory effect and eventually leads to programmed cell death. Variability in the inflammation index, attributed to surface charge, highlights the need for more specifically optimized dispersion methods to reduce inflammation and cell death in inhalation-based nanoparticles. This would reduce the toxic effects of NPs in downstream clinical applications.

One study investigated the effect of positively charged 50 nm MSNs suspended in BSA on the influx of neutrophils into the lungs and on the expression of pro-inflammatory genes 24 h post-MSN exposure in bronchioalveolar lavage (BAL) cells. A dramatic increase (22-fold) in the abundance of polymorphonuclear neutrophils and expression of interleukin-10 was observed compared to other types of plain and aminated synthesized MSNs, concluding that a more positively charged MSN increases the degree of inflammation within BAL cells [80]. Utilizing protein coatings to improve overall stability and neutralize the PSNs’ surface charge could potentially delay the premature release of materials from the pores of MSNs via electrostatic interactions between the amino groups on the MSNs’ surface. This method has opened a variety of doors for researchers to investigate the effect of surface charge and modify it with negatively or neutrally charged zeta potential surface functionalisation.

#### 2.2.5. Surface Functionalisation

Attachment of functional group(s) such as amine (-NH_2_), carboxyl (-COOH), thiol (-SH), and PEG enables PSNs to be used in targeted therapeutics, allowing proteins or receptors on target cells, effective uptake or recognition and subsequent unloading of therapeutic material into or near the target cells [48,81,82]. Studies indicate that functional groups also determine toxicity; for example, NPs functionalised with amine groups (compared to those functionalised with carboxyl or thiol groups) often exhibit increased cytotoxicity due to their positive charge, which can disrupt cell membranes and lead to cell death [83]. However, other studies have found that functional groups have made PSNs biocompatible. For example, quercetin-loaded amine-functionalised MSNs increased loading capacity, carboxyl functionalisation prevented particle agglomeration, and thiol functionalisation enabled efficient release. This shows the potential for effective oral drug delivery in the gastrointestinal tract [84]. PEGylation is frequently used to increase biocompatibility and decrease immunogenicity. PEG coatings can enhance drug and gene delivery by increasing systemic circulation time and reducing immune recognition [85,86]. A study compared PEGylated and non-PEGylated SiNPs and found that PEGylation reduced protein adsorption and immune activation while prolonging circulation time. However, PEGylation also reduced NP uptake by cancer cells, thereby balancing immune evasion and cellular uptake [87,88,89]. Targeted functionalisation, such as antibody-conjugated NPs, has been shown to increase selectivity for cancer cells, improve drug delivery, and reduce off-target effects [90,91,92]. However, some studies suggest that over-functionalisation by ligands/polymers may lead to unintended increases in recognition by opsonins, thereby increasing phagocytosis and reducing NP effectiveness [93,94,95]. Steric hindrance, which inhibits the interaction of the NP with its biological target [96]. Surface modification-dependent increased cellular internalization and pH-responsive release can effectively deliver therapeutics to the acidic tumour microenvironment, in contrast to unfunctionalised MSNs, which would circulate longer, increasing the risk of an immune response and the non-specific release of therapeutics into the bloodstream, potentially causing apoptosis in healthy cells [97]. Another interesting study to bypass the harsh barriers of the gastrointestinal tract used hollow silica NPs doped with quantum dots and functionalised with a two-part coating. A hydrophilic succinylated casein layer was designed to penetrate and effectively break through the mucosal layer, while a cationic cell-penetrating peptide concealed beneath the casein penetrates the epithelial layer to facilitate cellular uptake. The pharmacokinetic benefits from surface modifications resulted in a 5-fold increase in cell internalization and a 40% increase in the bioavailability of the loaded drug, paclitaxel [57]. Ultimately, the choice of surface functionalisation should be considered when designing SbD delivery vehicles: these should enhance bioavailability, target malignant cells, improve stability, facilitate cell membrane interactions, enhance uptake, and minimize downstream toxicity. However, balancing functionalisation for targeting and stealth properties remains challenging, as excess modification can lead to immune recognition and clearance. Future studies focused on various surface-engineering approaches will be critical for advancing PSN-based therapeutics for clinical applications.

#### 2.2.6. Agglomeration

PSNs are promising nanodrug carriers that require high dispersion and colloidal stability, as aggregation influences PSN internalization, biodistribution, and biological toxicity. As mentioned above, surface hydroxyl groups on PSNs, chemical modifications with proteins, polymer coatings, or phospholipid bilayer, can reduce agglomeration and maintain colloidal stability [16,98,99]. The agglomeration propensity of certain functionalised MSNs, such as low molecular weight PEG-coated MSNs, can exacerbate vascular injury by promoting larger areas of thrombosis, highlighting the need for a case-by-case evaluation based on the PEG molecular weight [100]. Similarly, amine-functionalised iron oxide NPs have been shown to cause cytotoxicity to HepG2 cells at higher concentrations, primarily due to agglomeration. Lower agglomeration reduced cytotoxicity, whereas higher levels increased it [83].

Another study observed a time-dependent effect on amorphous silica aggregates in human bronchial epithelial (HBE) cell cultures. When HBE cell cultures were exposed repeatedly to different sized (~2.5 µm vs. 100 nm) silica (aggregates) for two weeks and then allowed to recover (without amorphous silica exposure), silica aggregates of larger sizes (size ~2.5 µm) significantly affected the cell proliferation, and the release of IL-6, IL-8, and total glutathione at the end of both exposure cycles. In contrast, their nano-sized counterparts (<100 nm) induced more pronounced effects only at the end of the first exposure cycle. This study suggests that aggregates formed from larger silica NPs are toxicologically relevant and should be considered in risk assessment [101]. However, in another study, genotoxicity investigations using the comet and micronucleus assays on agglomerated mesoporous vs. non-agglomerated non-porous Swiss mice showed no evidence of genotoxicity in either group, indicating that agglomeration did not affect downstream toxicity [102].

On the other hand, under certain conditions, agglomeration can be advantageous. For instance, PSNs exhibit low bulk density and small particle size, challenging controlled flow toward target sites. Granulation of nanocarriers offers a partial solution by increasing bulk density, improving flow and enhancing delivery to the target site. Although this process decreases their pore volume and surface area, this reduction is negligible compared to the total specific surface area [16]. Future advances should focus on optimizing granulation parameters to maximize pore volume and therapeutic loading. Although this approach may compromise the surface area, it could offer benefits in terms of safety and minimizing toxicity, as the latter, in the case of PSNs, is strongly linked to the surface area of the pores that contain specific reactive silicic acid residues that generate toxicity by producing ROS, which can interact with the biological milieu.

#### 2.2.7. Crystallinity State

The toxicity and biological interactions of NPs critically depend on their physical state-crystalline or amorphous. Crystalline NPs have a highly ordered structure, which results in higher surface reactivity and, consequently, induces greater oxidative stress, DNA damage, lipid peroxidation, an inflammatory response, and cytotoxicity [103,104]. In comparison, amorphous NPs lack an ordered structure and are generally less toxic due to their increased solubility and decreased surface reactivity [105,106]. These differences impact fundamental biological processes, including ROS production, lysosomal stability, and immune recognition, affecting NP-induced cytotoxicity [107,108]. However, multiple studies have shown that they cause acute effects such as cytotoxicity via ROS generation. The toxicity of these NPs can be affected by the composition and synthesis method [109,110,111].

These observations indicate that crystallinity is a critical factor in determining the toxicity and biocompatibility of PSNs. Therefore, accurately identifying the physical state of NPs is crucial for ensuring their safety and efficacy.

In summary, standardized characterization of PSNs is needed to ensure reproducibility of the advanced material for scale-up manufacturing.

### 2.3. Assessment of Biocompatibility

To ensure the biocompatibility of PSNs, the biological interactions of these advanced materials within the body need to be studied extensively. Therefore, comprehensive functional toxicological assessments should include evaluations of acute and chronic toxicity, oxidative stress, inflammation, and organ-specific accumulation alongside analysis of protein corona formation, immunogenicity, and gene expression signalling pathways [112,113,114]. Biodistribution is also another essential aspect, as PSNs can enter the body through different routes of exposure. Incorporating these endpoints provides a better understanding of PSNs’ safety and functionality, supporting the development of safer biocompatible advanced nanomaterials.

#### 2.3.1. Cytotoxicity of PSNs

Cytotoxicity is an important factor in assessing NP safety and potential biomedical applications. Size [75], surface charge [115], shape [116], functionalisation [117], crystallinity [118], and exposure time [119], all play a role in determining the extent of cytotoxicity [120,121]. Cytotoxic effects can include membrane disruption [122], oxidative stress [123], mitochondrial dysfunction [124], apoptosis [125], necrosis [126], and inflammatory responses [127]. Studies have shown that different NPs can induce varied cytotoxic responses, underscoring the importance of comprehensive in vitro and in vivo evaluations.

The cytotoxicity of NPs can depend on the cell line used in toxicity assays, as different cell types may exhibit varying metabolic rates, uptake mechanisms, and antioxidant defences. According to a study comparing the effects of ZnO and SiO_2_ NPs, THP-1 cells were more sensitive to these NPs than L-132 cells [128], which correlates with the results published by Lanone et al. [129] who found variable sensitivity of human alveolar (A549) cells and macrophage (THP-1) cell lines to 24 different NPs. Another study found that 10 μg/mL of AgNPs exhibited varying levels of toxicity in cell lines. Ovarian cancer cells (A2780) were slightly more sensitive than breast cancer cells (MDA-MB 231), and both were more sensitive than MCF-7 cells [130].

The cytotoxicity of NPs can also be affected by a combination of physicochemical properties, cell-type specificity and exposure duration. The literature indicates that smaller, positively charged, and elongated NPs exhibit increased toxicity [75], whereas PEGylation and optimized surface functionalisation may reduce cytotoxicity [37,48]. Studies have demonstrated that the surface functionalisation of NPs significantly influences their cytotoxicity profiles. In some cases, non-functionalised NPs exhibit minimal or no cytotoxic effects, whereas functionalisation can introduce cytotoxicity depending on the nature of the surface modification. For instance, a study on mesoporous silica MCM-41 loaded with the anticancer drug, bicalutamide, showed that the empty MCM-41 nanocarrier did not induce toxicity. Cytotoxicity only increased once loaded with the drug. This indicates that without functionalisation, MSNs can be biocompatible and safe [131]. However, other studies have shown that MSNs without functionalisation are still cytotoxic. For instance, an investigation of MCM-41 and SBA-15 found that they were cytotoxic at most concentrations between 0.2 and 14 mg/mL to Caco-2 cells, causing membrane damage, ROS generation and increased apoptotic signalling [132]. Functionalisation of MCM-41 with essential oil components, eugenol and vanillin, showed greater cytotoxicity to HepG2 cells than bare MCM-41 after 48 h of exposure. It was suggested that due to the cationic nature and increased cell membrane interactions, cytotoxicity increased [133].

Cytotoxicity induced by PSNs must be well understood, both in bare and functionalised states, to prevent collateral toxicity and to ensure their safe and effective application in biomedicine.

##### Mechanism of Cytotoxicity-Oxidative Stress

Oxidative stress is a key mechanism underlying NP-induced cytotoxicity, driven by ROS generation and by cellular antioxidant defence systems [33]. ROS consist of a diverse range of highly reactive species, including hydroxyl radicals (•OH), superoxide anions (O_2_•^−^), and hydrogen peroxide (H_2_O_2_). Hydroxyl radicals are the most reactive and harmful and are highly capable of damaging cells. Superoxide anions and hydrogen peroxide are also less reactive than hydroxyl or carbon-centred alkyl radicals (R•) but can still contribute to oxidative stress and serve as precursors of stronger radicals [134,135].

NPs can enter cells and produce excessive ROS, leading to an imbalance of pro-oxidants to antioxidants [136]. This is achieved either by direct interaction with cellular components or by activating the mitochondrial pathway, which can result in oxidative damage to macromolecules such as lipids, proteins, and DNA [137]. One of the major consequences of ROS accumulation is lipid peroxidation, which can compromise cell membrane integrity, leading to increased permeability, loss of function, and cell death. Protein oxidation also inhibits enzymatic function, disrupting signalling cascades and leading to protein aggregation that may be cytotoxic [137,138,139].

Oxidative stress also damages DNA, forming DNA adducts, strand breaks, and mutations [140,141]. While this damage may activate several repair mechanisms, unresolved DNA lesions can trigger apoptotic pathways. The tumour suppressor protein p53 and the c-Jun N-terminal kinase (JNK) pathway are essential mediators of oxidative stress-induced apoptosis [142,143]. The activation of these pathways helps ensure that cells that are too damaged to function properly undergo programmed cell death rather than proliferate, thereby preventing potential mutations [144,145].

Enzymes such as glutathione peroxidase (GPx), catalase, and superoxide dismutase (SOD) are part of the cellular antioxidant defence system and contribute to the reduction of ROS-mediated damage [146,147,148]. Glutathione is a cellular antioxidant that quenches ROS and is also involved in detoxifying harmful metabolites [149,150]. Catalase breaks down hydrogen peroxide into water and oxygen, and superoxide dismutase catalyzes the dismutation of superoxide anions to produce oxygen and hydrogen peroxide [143]. However, when ROS production level exceeds the cell’s capacity to neutralize them with antioxidants, oxidative stress occurs, leading to cytotoxicity [151,152].

Several studies have shown that NPs, including PSNs, can induce ROS generation [153,154,155]. For example, a study demonstrated that SiNPs induced oxidative stress in HepG2 cells, causing apoptosis. They reported that smaller NPs (7–20 nm) induced greater oxidative stress than larger particles (50 nm), likely because of their higher surface area-to-volume ratio, which facilitates greater interactions with cells [37]. Another study showed similar results with MRC-5 fibroblast cells exposed to 62.5 μg/mL concentration of SiO_2_NPs. Increased levels of ROS were observed at 24, 48 and 72 h, confirming SiO_2_NPs induced ROS as a mechanism of cell survival [156]. Other NPs, such as iron oxide NPs (IONPs), have also been shown to generate ROS in vitro. For instance, IONPs triggered the regulated cell death process known as ferroptosis, characterized by lipid peroxidation and iron toxicity, in NRK-52E rat renal tubular epithelial cells. IONPs can promote oxidative injury and contribute to cell death via ferroptosis pathways [157]. Another study investigated the effect of IONPs on human microvascular endothelial cells. The study found that endothelial cell permeability increased following exposure to ROS-mediated oxidative stress induced by IONPs. The increase in permeability may affect vascular integrity and function [158].

Nevertheless, it should be noted that oxidative stress is not always the main mechanism of cytotoxicity induced by NPs. A recent study found that positively charged, PEI-modified MSNs, induced the accumulation of autophagosomes in HUVECs by inhibiting autophagosome maturation and hindering their fusion with lysosomes. This caused cytotoxicity in the absence of apparent ROS involvement [159]. Another study showed that in transformed human alveolar epithelial type 1-like cells, amine-modified polystyrene NPs induced severe membrane damage and altered cell adhesion, causing cell detachment and apoptotic cell death without ROS generation [160]. These studies further indicate that NP surface chemistry and charge distribution are key determinants of cytotoxicity, independent of oxidative stress pathways.

In general, oxidative stress is one of the main pathways by which NPs exert cytotoxicity. The relationship between ROS production, antioxidant defences, and resulting cellular damage emphasizes the need to assess oxidative stress to determine the safety of NPs for biological use.

To comprehensively evaluate these effects, various assays assess NP-induced cytotoxicity by measuring cellular metabolic activity, membrane integrity, and apoptosis. These include the 3-(4,5-dimethylthiazol-2-yl)-2,5-diphenyltetrazolium bromide (MTT) assay, lactate dehydrogenase (LDH) assay, trypan blue assay, caspase activity assay, annexin V/propidium iodide staining, alamar blue assay, cytokinesis block proliferation index and relative population doubling (Table 2). For more details, refer to Supplementary Materials. Combining oxidative stress assays with various cytotoxicity endpoints provides a broader view of cellular responses induced by NPs, which is crucial for their safety in biomedicine. Overall, these cytotoxicity assays provide valuable insights into the cellular responses induced by NPs; however, their reliability can be influenced by NP-assay interference [75]. Therefore, it is essential to evaluate potential interactions between NPs and assay components prior to use, to ensure accurate and reproducible results.

#### 2.3.2. Genotoxicity of PSNs

Genotoxicity is the ability of physical or chemical substances or materials to damage the genetic material within cells [161,162,163,164]. Genotoxicity testing is significant for assessing the safety of NPs, as it can determine whether materials, such as PSNs, can directly interact with DNA or disrupt cellular mechanisms that harm genetic material, leading to chromosome alterations or other modifications if DNA repair is impaired. It is a critical step in safety assessment for NPs or other xenobiotics that may not exhibit cytotoxic effects but can affect cellular health by inducing DNA damage, which may go unnoticed if genotoxicity is not assessed [165]. Similar to cytotoxicity, the genotoxic potential of NPs is influenced by several physicochemical properties such as size, shape, surface charge, crystallinity, and functionalisation [166]. Understanding these mechanisms is critical for evaluating NP safety and mitigating potential risks in biomedical applications.

##### Physicochemical Dependent Properties Affecting Genotoxicity

Physicochemical characteristics of NPs play an essential role in their genotoxic potential. Size-dependent effects have been widely observed, with smaller NPs showing higher nuclear translocation and stronger interactions with DNA [167]. In a study on AuNPs of different sizes (5, 20, and 50 nm), 5nm AuNPs caused dose-dependent DNA damage in HepG2 cells after 24 h of treatment; however, 20 and 50 nm AuNPs did not induce evident DNA damage. The 5 nm AuNPs also caused cell cycle arrest and increased ROS production [168]. Another study compared different sizes of amorphous SiNPs (11 nm, 34 nm, 248 nm) in 3T3-L1 mouse fibroblasts. Chromosomal aberration induction occurred only with 34 nm particles, illustrating the importance of optimum particle size and genotoxicity [169,170].

The shape of NPs is another important factor that dictates genotoxicity assessment. For instance, elongated NPs, such as nanorods and nanowires, are reported to mechanically stress chromosomes, causing mitotic errors [171]. Sphere-, rod- or wire-shaped TiO_2_NPs have been shown to induce DNA damage in the Caco-2/HT-29 cell model with the comet assay. The greatest genotoxicity was observed with rod-shaped NPs, confirming their interference with mitotic processes [172]. The surface charge of NPs plays a key role in their genotoxicity by influencing their interactions with DNA [173]. Positively charged NPs interact more strongly with the negatively charged DNA backbone, leading to DNA condensation and interference with transcription [174]. A study demonstrated that amine-functionalised SiNPs showed a stronger binding affinity to DNA, leading to greater genotoxicity than carboxyl-functionalised NPs, which had weaker electrostatic interactions with genetic material [75,175]. Similarly, positively charged PLGA NPs induced genotoxicity in 16HBE14o human bronchial epithelial cells as assessed by the micronucleus and comet assays; however, negatively charged or neutral NPs showed no genotoxicity. Results showed chromosomal aberrations without primary DNA damage [176]. This correlation emphasizes the importance of surface charge in NP-induced genotoxicity.

Crystallinity of NPs also significantly influences genotoxicity. Crystalline NPs can generate higher ROS than amorphous ones; excess ROS production can induce oxidative stress, leading to oxidative DNA injury and micronuclei formation [177]. A study showed that anatase TiO_2_ NPs typically induce DNA strand breaks and chromosomal damage, whereas rutile TiO_2_ NPs are predominantly nongenotoxic in vitro [178]. On the other hand, a different study demonstrated that TiO_2_-A NPs, mainly anatase, were cytotoxic and had little oxidative effect on both cell lines, whereas TiO_2_-B NPs, mainly rutile, induced genotoxic and oxidative effects in both cell lines, contradicting the previous study [179]. Research has also demonstrated that amorphous NPs can be genotoxic, like crystalline NPs. For instance, synthetic amorphous silica showed a dose-dependent genotoxic (0.01 to 150 µg/mL) effect in the mouse lymphoma assay, which detects genetic mutations [180]. Crystalline and amorphous niobium oxide NPs (NINPs) also showed genotoxicity in CHO-K1 cells. The crystalline NINPs exhibited high genotoxicity after 4 h of exposure with increased micronuclei and chromosomal instability, whilst amorphous NINPs demonstrated lower genotoxicity with less micronuclei formation after 24 h of exposure [181]. This highlights the significance of NP crystallinity in determining their genotoxic potential.

##### Mechanisms of Genotoxicity

NPs induce genotoxic effects through multiple mechanisms. One major mechanism is through oxidative stress. Oxidative DNA alterations, such as breaks, base oxidation, and chromosomal instability, are caused by excessive ROS [182]. Several NPs have induced oxidative stress, leading to genotoxicity. For example, AgNPs have been shown to induce DNA damage by generating ROS. They induced DNA damage via an oxidative stress mechanism after 30 min of exposure in human embryonic epithelial cells using a modified comet assay [183]. Another study showed the genotoxicity of titanium dioxide nanoparticles (TiO_2_NPs) in human epidermal cells, identifying their potential to induce DNA damage, possibly by oxidative stress. The results indicated that TiO_2_NP exposure led to ROS upregulation, as evidenced by oxidatively modified DNA, SSBs (single-strand breaks), and DSBs (double-strand breaks). Consequently, the upregulation of oxidative stress-associated biomarkers was also observed, underscoring the role of ROS generation as a key factor in TiO_2_NP-mediated genotoxicity [184]. Tin-loaded MSNs also induced significant ROS generation in MCF-7 cells at a concentration of 5 μg/mL after 6 h. These MSNs also caused genotoxic effects in CHO cells (2–8 μg/mL, 24 h), including chromosome aberrations, micronucleus formation, and a reduction in mitotic index [185]. This emphasizes the role of oxidative stress as the major driver of NP-mediated DNA damage, which, depending on dose and duration of exposure, could potentially lead to mutagenesis and carcinogenesis.

In addition to oxidative stress, direct DNA interaction also contributes to NP-induced genotoxicity. It has been reported that some NPs can enter the nucleus and interact with genetic material, resulting in DNA strand breaks, chromatin condensation, and transcriptional interference [164,165,184]. For example, IONPs were shown to form complexes with DNA that could also intercalate between base pairs. This direct interaction caused chromosomal aberrations, micronucleus formation, and DNA damage in vitro and in vivo [186]. Also, SiO_2_ NPs (10–500 μg/mL) caused a concentration-dependent increase in DNA strand breaks, reaching a 13-fold increase at 300 μg/mL after 24 h of exposure, without inducing ROS generation in human peripheral blood mononuclear cells [187]. Similarly, another study demonstrated that SiNPs induced H2AX phosphorylation in spermatocyte cells of mice, leading to DNA double-strand breaks in a dose-dependent manner across concentrations of 6.25 μg/mL to 50 μg/mL [188].

Another mechanism of genotoxicity involves interference with mitosis. In this mechanism, microtubules that move chromosomes during cell division (spindle microtubules) are disrupted by NPs, leading to chromosome missegregation and aneuploidy [189,190,191]. For example, it has been shown that microtubule networks can be altered by highly water-dispersible single-walled carbon nanotube (SWCNT) nanosystems, and their effects on mitotic spindles have been studied, leading to the conclusion that these NPs may induce chromosomal instability. In human airway epithelial cells exposed to SWCNT, the authors noted duplicated and fragmented centrosomes, disrupted mitotic spindles, and abnormal aneuploid chromosome numbers [192]. Other engineered nanomaterials, such as copper-based metal–organic frameworks, have demonstrated the ability to promote tubulin polymerization and disrupt microtubule dynamics. This can interfere with the formation and function of the mitotic spindle, resulting in an abnormal mitotic spindle [193,194].

There have also been extensive studies on inflammation-driven genotoxicity, driven by NPs’ ability to elicit inflammatory responses, leading to subsequent oxidative stress and DNA damage [195]. Senapati et al. [196] showed that ZnONPs could induce the upregulation of pro-inflammatory cytokines such as interleukin 1β (IL-1β) and TNF-α in THP-1 macrophages, which subsequently may contribute to oxidative stress-generated genotoxicity. It has been shown that inflammatory cytokines can cause DNA damage by generating reactive nitrogen species, such as nitric oxide and peroxynitrite. These reactive species can induce oxidative damage, leading to genomic instability. Consequently, chronic inflammation is a significant driver of genomic instability [197]. Oesophageal epithelial cells are another example of how chronic inflammation can lead to malignant transformation by inducing genomic instability. Studies suggest prolonged exposure to inflammation can disrupt genomic integrity, increasing the risk of malignant transformation over time [198]. Such a mechanism is highly relevant to the development of NPs for long-term biomedical applications, given the potential for repeated exposure to lead to irreversible inflammatory responses and cumulative DNA damage.

Various genotoxicity assays (Table 3) can be used to assess different types of DNA damage including the cytokinesis block micronucleus (CBMN) assay for detecting chromosomal breakage or loss, the comet assay for measuring DNA strand breaks, the chromosome aberration test for identifying structural chromosomal alterations, γ-H2AX assay for detecting DNA double-strand breaks and the Ames Test for evaluating mutagenic potential in bacteria. For more details, refer to Supplementary Materials.

#### 2.3.3. Gene Expression Profiles

Gene expression profiling is a useful tool for examining the molecular mechanisms underlying PSN-induced toxicity. Exposure to different NPs alters intracellular signalling cascades and gene expression patterns, highlighting the pathways involved in oxidative stress, inflammatory response, apoptosis and DNA damage [199].

The p53 pathway plays a central role in the cellular stress response to NPs by regulating gene expression responsible for DNA repair, apoptosis, and cell cycle arrest [200,201]. Upstream regulators of p53 include DNA damage sensors such as ATM (ataxia-telangiectasia mutated) and ATR (ataxia-telangiectasia and Rad3-related) kinases, which phosphorylate and stabilize p53 in response to DNA damage [202]. Other upstream regulators include murine double minute 2 (MDM2), an E3 ubiquitin ligase that normally targets p53 for proteasomal degradation, which is inhibited under stress, allowing p53 to accumulate [203]. Once p53 is activated, it acts as a transcription factor, regulating a wide range of downstream target genes that influence cellular fate [204].

Following DNA damage, p53 up-regulates the expression of p21 (CDKN1A), a cyclin-dependent kinase (CDK) inhibitor, which causes cell cycle arrest at the G1/S checkpoint to allow time for DNA repair [205,206]. If the damage is beyond repair, p53 activates apoptosis by increasing the expression of pro-apoptotic genes such as BCL-2-associated X protein (BAX), p53 upregulated modulator of apoptosis (PUMA), and phorbal-12-myristate-13-acetate-induced protein 1 (NOXA), causing mitochondrial outer membrane permeabilisation (MOMP) and caspase activation, ultimately resulting in programmed cell death [207,208]. Furthermore, p53 regulates senescence, a permanent state of cell cycle arrest, by modulating the p21/p16INK4a and pRB pathways, thereby inhibiting aberrant cell proliferation [209,210].

Nanoparticles have been shown to activate the p53 pathway through mechanisms of oxidative stress and DNA damage, leading to varied cellular responses depending on their physicochemical properties [211]. Wierzbicki et al. [212] investigated the effects of graphene oxide nanoplatelets and graphite NPs on glioma cell lines with varying p53 status. They demonstrated that NPs diminished the angiogenic potential of glioma cells, with p53 status-dependent effects. In wild-type p53 cells, NPs inhibited NF-kB activity, leading to lower levels of inflammatory cytokines and oxidative stress. Contrarily, in mutant p53 cells, NF-kB signalling remained active, leading to persistent inflammation and cellular stress, highlighting the role of p53 in mitigating NP-triggered toxicity. Further evidence of p53 pathway activation was observed in primary pulmonary epithelial cells; prolonged exposure to TiO_2_NPs was accompanied by elevated expression of key apoptotic proteins, including BAD, BAX, and phospho-p53 [213]. Similar results were observed in MCF-7 cells, where MSNs coated with polydopamine and loaded with umbelliprenin activated the TP53 pathway. Treatment with 30–45 μg/mL for 48 h resulted in approximately a 3-fold increase in p53 gene expression, with the activation associated with apoptosis [214].

**Figure 3:**
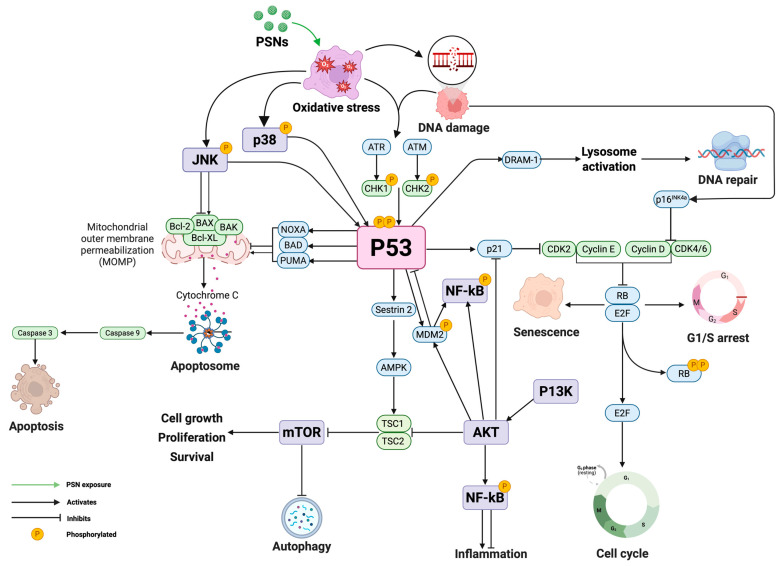
PSN-induced activation of the p53 signalling pathway and its crosstalk with mTOR, PI3K/AKT, NF-kB, and MAPK pathways (Created in BioRender. Patel, T. (2025) https://BioRender.com/dwxczus). This schematic diagram illustrates the potential p53 signalling pathway in response to PSN-induced oxidative stress and DNA damage. Oxidative stress from PSNs activates JNK and p38 MAPK pathways, which phosphorylate p53, enhancing its transcriptional activity. Simultaneously, DNA damage triggers ATM/ATR kinases, leading to CHK1/CHK2 phosphorylation and p53 stabilization. Once activated, p53 regulates multiple cellular outcomes, including cell cycle arrest, apoptosis, senescence, and DNA repair. Cell cycle arrest occurs through p21 (CDKN1A) upregulation, which inhibits CDK2, and Cyclin E, preventing G1/S transition. DNA damage activates p16INK4a/RB pathway, while p53-induced DNA repair is facilitated via DRAM-1-mediated lysosomal activation. If the damage is irreparable, p53 induces apoptosis by upregulating pro-apoptotic genes (NOXA, BAD, PUMA, BAX, and BAK), leading to MOMP, cytochrome c release, and activation of caspase 9 and caspase 3. Additionally, p53 interacts with mTOR signalling, where Sestrin 2 activates AMPK, inhibiting mTOR (via TSC1/2) and autophagy. The PI3K/AKT pathway antagonizes p53 by activating MDM2, leading to p53 degradation, while also promoting cell survival and proliferation. Furthermore, AKT activation stimulates NF-kB, enhancing inflammatory responses and counteracting p53-mediated apoptosis. This intricate interplay between p53 and other pathways determines cellular fate, balancing repair, survival, or cell death in response to PSN exposure.

The p53 pathway interacts with various signalling pathways that mediate cellular homeostasis and stress responses. Figure 3 illustrates this complex network, highlighting crosstalk between the p53 pathway and other signalling pathways. The NF-kB pathway is a key pathway that interacts with p53; it is involved in inflammation and immune responses [215,216]. While p53 mainly induces apoptosis and cell cycle arrest following DNA damage, the activation of NF-kB can oppose this signalling by promoting cell survival and inflammatory pathways [217,218]. This relationship is intricate because NF-kB can inhibit p53 via MDM2 upregulation, leading to p53 degradation, but p53 can also inhibit NF-kB activity by competing with transcriptional coactivators like p300/CBP [219,220]. Previous research has demonstrated that NPs can alter p53 and NF-kB expression. For instance, silica NPs ranging from 50 to 200 μg/mL concentration caused ROS, mitochondrial depolarization and apoptosis after 24 h of exposure in HUVEC cells. It was demonstrated that silica NPs induced cell damage by oxidative stress via p53, NF-kB, and JNK signalling pathways [221]. This suggests that silica NPs, including PSNs, can induce cellular stress responses through oxidative stress-mediated signalling pathways, leading to mitochondrial dysfunction and apoptosis.

Another important pathway that modulates p53 activity is the MAPK pathway, which comprises three components: extracellular-signal-regulating kinase (ERK1/2), stress-activated protein kinase/c-Jun-N-terminal kinase (JNK) and p38 [222]. p38 MAPK activation upon cellular stress promotes p53 phosphorylation at specific serine and threonine residues, thereby increasing its stability and transcriptional activity. This is particularly relevant in responses to oxidative stress and in DNA damage repair [223,224]. In specific cellular contexts, ERK may stabilise or promote the degradation of p53 [225,226]. MSNs loaded with resveratrol (plant polyphenol) can modulate ROS levels and activate the p38-MAPK/p53 signalling pathway, leading to increased apoptosis and autophagy in hypertrophic scar fibroblasts [227]. Also, another study demonstrated that silica NPs activated p53 by the JNK pathway activation, resulting in p53 acetylation and cytoplasmic localization, leading to mitochondrial cytochrome c release and apoptosis [228]. These results highlight the significance of the MAPK pathway as a critical regulator of cellular stress responses through its interaction with p53.

The p53 tumour suppressor protein also interacts with the autophagy pathway and has a dual role depending on cellular stress levels [229,230]. In response to mild stress, p53 can promote the transcription of autophagy-related genes, such as Atg, DRAM, and Sestrin1/2, thereby facilitating the clearance of damaged cellular organelles [231,232]. However, mutant p53 states can promote increased ROS production during autophagy, indicating that NF-kB acts as a negative regulator of autophagy and further highlighting the crosstalk between the two pathways [233,234].

In addition, the phosphoinositide 3-kinase (PI3K)/AKT/mammalian target of rapamycin (mTOR) pathway is closely related to p53 in determining cell survival or death [235]. Dysregulation of the PI3K/AKT pathway often occurs following p53 inactivation. On the other hand, p53 downregulates mTOR signalling by upregulating the AMPK pathway, thereby restricting cell proliferation under stress [236,237]. Recent research has demonstrated that MSNs can modulate the P13K/AKT/mTOR signalling cascade, promoting cell proliferation and tissue repair but also preventing apoptosis [238,239].

NPs can also modulate NF-kB downregulation in p53 wild-type glioma cells, thereby reducing inflammatory cytokine expression [212]. In contrast, p53 and NF-kB can cooperate to regulate pro-inflammatory responses in human macrophages, thereby modulating the expression of interleukin-6 (IL-6), interleukin-8 (IL-8), and growth-regulated oncogene alpha (GRO-α). The cytokines are known to induce neutrophil migration and immune activation, suggesting that immune cell-specific responses can be induced by NF-kB modulation via NPs [217].

The surface chemistry of NPs has been found to play a role in NF-kB activation. Research showed that the activation of the NOD-like receptor protein 3 (NLRP3) inflammasome and the production of IL-1β were influenced by the surface reactivity of carbon nanotubes. Polyetherimide induced heavy cationic functionalisation and increased secretion of the inflammatory mediators IL-1β, TGF-1, and PDGF-AA in BEAS-2B and THP-1 cell lines [240]. This suggests that NPs with strong cationic functionalisation exhibit greater immunogenicity and inflammation due to strong interactions with cells, activating the NLRP3 inflammasome and leading to the subsequent secretion of cytokines. Therefore, understanding how different NP properties influence gene expression changes is essential for safer and more effective nanomedicine applications.

These interactions highlight that p53 does not act alone but is embedded in a larger signalling network that regulates cellular responses to stress, damage, and metabolic fluctuations. Insights into these coupled networks will provide an important basis for the engineering of therapeutic interventions targeting p53 and its related pathways in disease.

#### 2.3.4. Protein Corona

**Figure 4:**
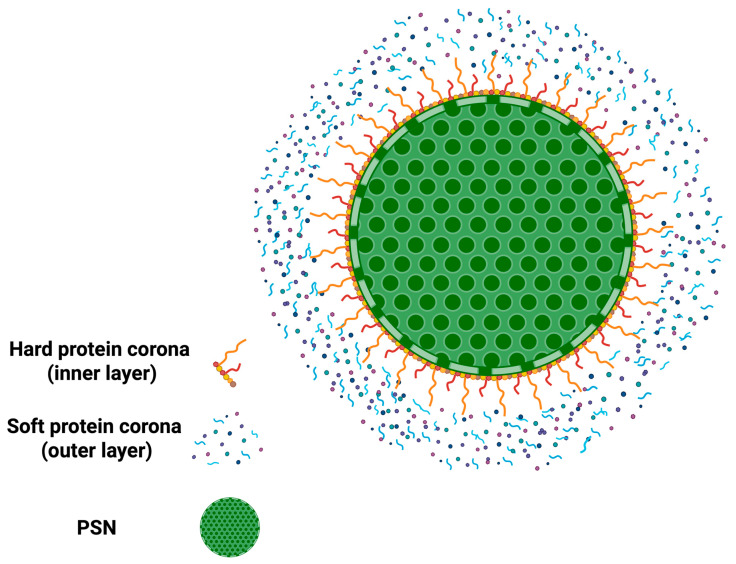
Structural organization of protein corona on PSNs (Created in BioRender. Patel, T. (2025) https://BioRender.com/fcoz305). A schematic diagram of protein corona formation on the surface of PSNs. In the middle is the core of the NP, surrounded by the inner layer, which represents the hard protein corona and the outer layer, which represents the soft protein corona.

The protein corona is a dynamic layer of proteins that surrounds NPs upon exposure to biological fluids. Protein corona formation has significant implications in nanomedicine, as it modifies NPs’ biological identity and often affects (facilitates or restrains) cell targeting, impacting cellular uptake and in vivo biodistribution [241,242,243]. The protein corona comprises two distinct layers: the hard corona, representing a tightly bound inner layer of proteins with a high affinity for the NP surface, and the soft corona, composed of loosely associated proteins that dynamically exchange with the surrounding biological environment (Figure 4) [244,245,246,247]. For PSNs, this is especially relevant, as the corona would strongly affect their stability, cellular interactions, toxicity, and targeting capabilities. Incubation of SiNPs with human plasma identified over 300 different proteins in the corona [248,249]. The findings indicated that hard corona proteins remain bound to NPs for several hours, while soft corona proteins exchange within minutes, highlighting the dynamic nature of the protein corona in biological fluids.

Transferrin ligands on silica NPs can be masked by plasma proteins, hindering receptor-mediated endocytosis and resulting in non-specific uptake [250,251,252], thereby compromising targeting specificity. Conversely, PEGylated polymeric NPs that adsorbed apolipoproteins reduced rapid clearance by immune cells and prolonged blood circulation times, suggesting that the appropriate corona composition may improve NP stability in vivo [253]. However, protein corona formation can also activate the immune system, triggering complement activation and opsonisation, which leads to faster clearance from circulation [254,255,256].

Albumin is the most abundant protein found in NP coronas, including silica [257,258,259]. While association with some proteins, such as albumin, increases the circulation time of NPs by preventing aggregation and reducing macrophage recognition [259,260,261] while others, including complement proteins 3 (C3) and 4 (C4), are associated with increased immune clearance [262,263].

Among others, immunoglobulins (IgG, IgA), fibrinogen, transferrin, and apolipoproteins (ApoA1, ApoE) are commonly identified [257,264]. Opsonisation by immunoglobulin adsorption has been shown to promote enhanced phagocytosis, while apolipoproteins modulate NP transport across biological barriers, including the BBB [265,266,267]. Specifically, MSNs in biological fluids can adsorb plasma proteins, which can either reduce their opsonisation and recognition by phagocytic cells or promote phagocytosis, affecting their effectiveness as drug delivery systems [268,269]. Therefore, there is a need for surface engineering strategies to control immune recognition and improve therapeutic efficacy by coating MSN surfaces with polymers, such as PEG, that can diminish protein adsorption and opsonisation, and extend the circulation half-life of MSNs, resulting in higher drug delivery efficiency [87,270].

Likewise, due to their surface charge, positively charged lipid-based NPs (cationic liposomes) have been shown to bind efficiently to plasma proteins during drug delivery [271,272], thereby labelling the NPs for opsonisation and elimination by phagocytic cells. These interactions illustrate how NP surface properties dictate biological response and emphasize the need for a design that takes therapeutic efficacy and immune system interactions into consideration [273,274,275].

Understanding this protein-NP interaction is important for maximizing the efficacy of these NPs in drug delivery and imaging applications. Multiple analytical techniques are employed to characterize the protein corona, including protein composition, binding affinities and structural properties of the associated proteins including liquid chromatography tandem mass spectrometry (LC-MS/MS) [276,277], sodium dodecyl sulphate-polyacrylamide gel electrophoresis (SDS-PAGE) followed by Western blotting [257,278] and dynamic light scattering (DLS) that registers the alterations of NP dimensions triggered by the crown buildup which serve as indirect proof of protein adhesion. It is typically used in conjunction with zeta potential determination to gain insight into how surface charge is altered by corona formation [279,280].

Despite differences in protein corona composition, a general trend shows that positively charged NPs, including silicon dioxide (SiO_2_) NPs attract more proteins than negatively charged NPs [281], indicating that surface charge influences protein adsorption [282]. The influence on protein corona is not only charge-dependent but also functionalisation-dependent. PEGylation has been shown to decrease protein adsorption, leading to less bound protein on the surface [270], which illustrates the compromise between stability and cellular recognition in NP drug delivery [283]. Understanding protein corona formation and composition is essential for optimizing the performance of PSNs in biomedical applications.

#### 2.3.5. Immunogenicity of PSNs

Several physicochemical properties, such as size, surface charge, shape, and surface functionalisation, determine the ability to induce an immune response, or immunogenicity, a factor that can significantly affect the overall efficacy and safety of NPs [284]. One of the most pronounced immune mechanisms influencing the fate of PSNs is mediated by the complement system, which may contribute to their clearance, immune recognition, and subsequent inflammatory responses. Thus, understanding the mechanisms of complement activation is essential to enable their suitable tuneability for medical applications [285,286].

**Figure 5:**
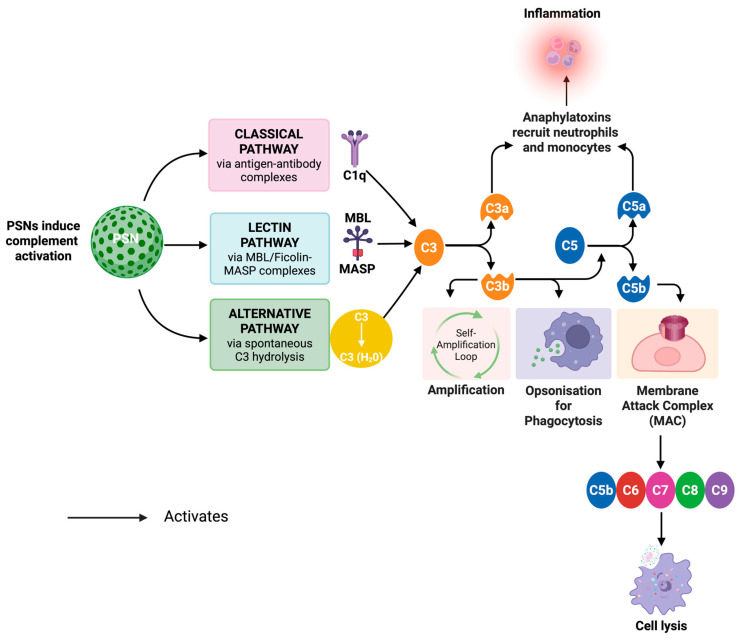
Complement activation pathways induced by PSNs (Created in BioRender. Patel, T. (2025) https://BioRender.com/i2x0h8c). This figure illustrates the activation of the complement system by PSNs via three distinct pathways: the classical, lectin, and alternative pathways. The classical pathway is triggered by antigen–antibody complexes involving C1q, while MBL and MASP activate the lectin pathway. The alternative pathway is initiated through spontaneous hydrolysis of C3 into C3(H_2_O). Activation of C3 leads to the generation of C3a and C3b, where C3a functions as an anaphylatoxin that recruits neutrophils and monocytes, contributing to inflammation, while C3b facilitates opsonisation for phagocytosis. Further activation of C5 results in the formation of C5a, another potent inflammatory mediator, and C5b, which initiates MAC formation. The MAC, composed of C5b, C6, C7, C8, and C9, leads to cell lysis. Additionally, a self-amplification loop of C3 enhances the complement response. This schematic highlights the immune-modulatory effects of PSNs, demonstrating their potential to trigger complement activation, which may influence their biocompatibility and therapeutic applications.

The complement system consists of more than 30 plasma proteins that recognize pathogens, promote inflammation, and stimulate phagocytosis; it is a critical component of the innate immune response. It is activated by three pathways (Figure 5): the classical pathway, the lectin pathway, and the alternative pathway [287]. The three pathways converge at the cleavage of C3 to generate C3b, a key opsonin that enhances immune cell recognition, and C5b, which initiates the assembly of the membrane attack complex (MAC) [288]. Thus, the activated pathways may play an important role in the processing of PSNs within the body, influencing circulation time, clearance, and toxicity [289,290]. A study demonstrated that dextran-coated NPs activated complement primarily via the alternative pathway in human serum [290]. Another study also showed that complement activation led to the deposition of C3b on NP surfaces. This opsonisation facilitates recognition, movement, and clearance by immune cells, specifically macrophages and dendritic cells [291]. This has implications for PSN biodistribution, as complement activation can result in rapid systemic clearance, reducing their use as drug delivery carriers or for imaging applications [292,293].

The role of complement proteins C1–C9 in NP recognition varies depending on the physicochemical properties of NPs. C1q initiates the classical pathway by binding to immunoglobulin-coated NPs or other specific ligands on their surface [286]. Studies found that IgG-enriched protein coronas around NPs showed strong C1q binding and enhanced classical pathway activation [95,258]. Alternatively, the lectin pathway is initiated by mannose-binding lectin (MBL), ficolin, and MBL-associated serine proteases (MASPs), which recognize carbohydrate patterns on NP surfaces. It has been shown that some gold and silica NPs functionalised with sugar moieties can activate this pathway [294,295,296]. Most NPs activate complement through the alternative pathway, driven by spontaneous C3 hydrolysis and the deposition of C3b onto the NP surface. Research showed that, compared with low- or non-charged particles, cationic NPs were associated with stronger complement activation [293]. Similarly, another study showed that gold NPs passivated with PEI, a positively charged polymer, showed enhanced complement activation compared to other polyelectrolyte ligands [297].

After the complement cascade is activated, important downstream effects include phagocytosis and the formation of MAC. C5 convertase cleaves C5 into C5a and C5b, with C5a playing an important role as an inflammatory mediator, recruiting neutrophils and monocytes to areas of immune activation. C5b initiates MAC (C5b–C9) formation and creates pores in microbial and cellular membranes, leading to induced damage and inflammatory toxicity when overactivated by NPs. Excessive MAC formation induced by NPs triggered hypersensitivity reactions, inflammation, and complement-mediated toxicity, especially during intravenous NP administration [298,299,300].

Complement activation affects NP function in two distinct ways. On the one hand, it clears the NPs, preventing the accumulation of foreign materials in circulation. This is beneficial for vaccine adjuvants and antimicrobial NPs, in which complement activation can promote pathogen clearance and activate the immune response [286]. On the other hand, excessive complement activation is detrimental to NP drug delivery systems as it reduces their circulation time [301]. For example, the opsonisation of transferrin-functionalised SiNPs reduced targeting efficiency, as complement proteins masked transferrin ligands, thereby preventing their uptake by cancer cells [251].

Surface functionalisation is one of the most used strategies to modulate complement activation while maintaining NP function. One approach is PEGylation; NP PEGylation can reduce complement activation by reducing protein adsorption. This steric hindrance reduces interactions with other molecules and aggregation by creating a conformational cloud around PEG chains. This hydrophilic barrier effectively masks the NP surface from recognition by positive complement proteins [301]. Nonetheless, even PEGylated NPs may accumulate C3b molecules, leading to immune recognition after extended circulation time [87,302]. It is suggested that zwitterionic coatings mimic cell membranes and thus lead to lower complement activation than PEGylation [303]. Another strategy focuses on biomimetic modifications, in which CD47-functionalised NPs present a native “self” signal to antagonize complement-mediated phagocytosis [304,305]. In addition to surface engineering, pre-coating NP surfaces with either albumin or apolipoproteins has become a strategy to protect against excessive complement activation while maintaining functional transport properties [306,307]. It was reported that NPs precoated with albumin reduced C3b deposition, thereby prolonging circulation and improving biodistribution [308].

NPs also interact with antigen-presenting cells (APCs) and T and B cells to modulate adaptive immune responses. The potential of some NPs to act as haptens can stimulate T-cell-dependent immune responses by facilitating antigen uptake and dendritic cell presentation [309,310]. This, however, may result in antibody production against NPs, potentially affecting their long-term stability and biodistribution [293]. Moreover, NP-mediated inflammasome activation has been associated with various chronic inflammatory conditions. Evidence for NLRP3 inflammasome activation leading to IL-1β secretion and inflammatory toxicity from metal oxide NPs has been demonstrated in HepG2 and THP-1 macrophage models [241,311].

Overall, understanding the immunogenicity of NPs, particularly their capacity to induce complement activation, is complex and depends on multiple physicochemical features [284]. Complement activation could potentially promote immune clearance and stimulate the immune response in vaccine applications, but excessive activation can cause inflammation, hypersensitivity reactions, and rapid clearance, which is detrimental in NP drug delivery applications [312]. Understanding and modulating these immune interactions with novel surface modifications, biomimetic coatings, and complement inhibitors should be further developed to address immune evasion and immune compatibility of NPs, thereby maintaining NP materials’ functionality and biocompatibility in future clinical applications [293,313,314].

### 2.4. Facilitation of Toxicity: Exposure Routes and Relevant Models

**Figure 6:**
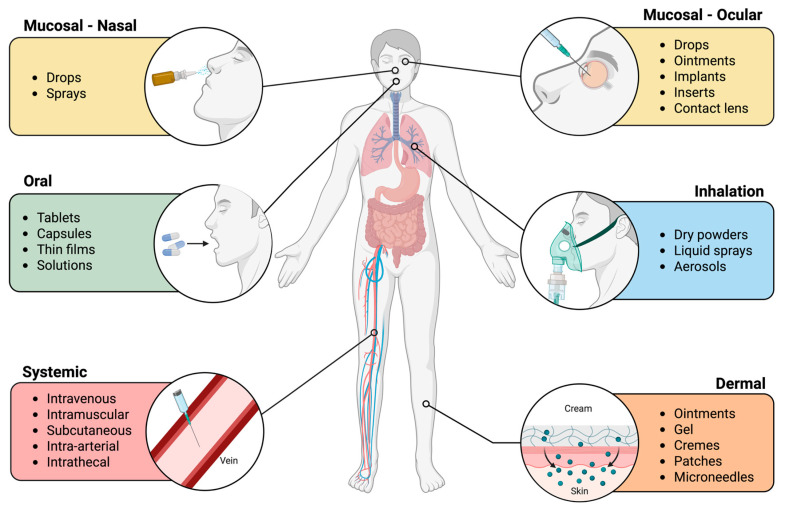
Routes of NP exposure and administration (Created in BioRender. Patel, T. (2025) https://BioRender.com/d8hm9lr). This figure illustrates the various routes of NP exposure, including mucosal (nasal and ocular), oral, inhalation, systemic, and dermal pathways, each with specific formulations designed for targeted delivery.

The biological effects of NPs depend mainly on their exposure route, which determines their biodistribution, metabolism and elimination [315,316]. Hence, NPs can be administered through respiratory (inhalation) [317,318], dermal (skin) [319], oral (mouth and gut) [320,321,322,323], systemic (injection) [114,324] and mucosal (membrane entry and into the bloodstream) [325,326] exposure, each presenting different interactions with biological environments (Figure 6). Due to the complexity of NP interactions across distinct biological environments, it is essential to use diverse cell lines that best represent these exposure routes [130,327], thereby narrowing the gap between in vitro and predicted in vivo effects.

#### 2.4.1. Inhalation

Inhalation is a key exposure pathway, especially for engineered nanomaterials, which are often manufactured and processed in occupational environments [328,329]. The particles are small enough to invade the lungs as far as the alveolar space, translocate into the blood and target secondary organs such as the liver, kidneys and brain, raising concerns about their systemic toxicity. Oxidative stress, inflammation, and pulmonary toxicity may be induced by inhaled NPs [330]. Human monocyte-derived THP-1 cells and 16HBE14o-(16HBE) human bronchial epithelial cells have been used in an in vitro model of airway barrier properties to test the impact of NPs on airway epithelial cells [331]. Additionally, Calu-3 human bronchial epithelial cells have been employed to mimic bronchial epithelial barrier function, mucus production, and epithelial repair, providing insights into how NPs may affect respiratory health [332,333].

#### 2.4.2. Dermal

Dermal exposure to NPs can occur through multiple pathways, such as cosmetics, transdermal drug delivery systems, and wound dressings [319,334,335]. While the stratum corneum constitutes a natural barrier, prolonged exposure or disruption in skin integrity can allow NPs to penetrate more deeply into the skin and potentially enter systemic circulation [336]. Factors like skin diseases, wounds, or mechanical damage may increase the likelihood of deeper NP penetration [337]. TiO_2_NPs and ZnONPs, used in sunscreens, have generated interest due to their potential to induce oxidative stress in skin cells.

These can induce oxidative stress and inflammation in skin cells. ZnONPs were found to inhibit cell proliferation and cause DNA damage in human keratinocytes (HaCaT cells) [338]. However, extensive systemic absorption has yet to be conclusively demonstrated, and some researchers have suggested that the risk of dermal exposure could be exaggerated [339].

#### 2.4.3. Oral

Oral exposure is another common route for NPs, intentional through NP-based drug formulations [320,340]. The GIT can influence the fate of ingested NPs by exposing them to digestive enzymes, acidic pH, and the microbiota, which can modify the physicochemical properties of NPs [341,342]. NP uptake by intestinal epithelial cells occurs via transcellular or paracellular pathways; NPs pass through the intestinal barrier and enter the blood circulation, where they accumulate in organs such as the liver and spleen [343,344]. As the liver plays a central role in NP clearance and detoxification, HepG2 cells have thus been used extensively to assess hepatic metabolism and cytotoxicity of ingested NPs [345,346]. In addition to HepG2 cells, Caco-2 human colorectal adenocarcinoma cells are extensively used to model the intestinal epithelium for studying NP absorption and transport mechanisms [347]. Furthermore, another human colorectal adenocarcinoma-derived cell line, HT-29 line, is used to study mucus-secreting characteristics and the effects of NPs on intestinal mucus production [348,349].

#### 2.4.4. Systemic

Intravenous and systemic administration are other widely used exposure routes for NPs in biomedical applications [7,350]. However, NP clearance by the circulating immune cells [345] encourages the progress of long circulating nanocarriers, typically comprising stealth coatings such as PEG to minimize immune identification [87,351]. Despite these innovations, concerns persist regarding NP-induced immunogenicity, thrombosis, and organ accumulation following intravenous administration [352,353]. Commonly used cells for testing genotoxicity caused by NPs are human lymphoblastoid TK6 cells, which have an intact p53 tumour suppressor gene, enabling evaluation of responses to DNA damage due to p53’s well-characterized role in mediating cell cycle progression and apoptosis in response to genotoxic stress. This is particularly relevant, as cells carrying mutated or deficient p53 can respond differently to DNA damage, potentially leading to misleading results in genotoxicity assessments.

#### 2.4.5. Mucosal

Another potential route of NP uptake is mucosal exposure, including ocular and nasal absorption [354,355]. PSNs have emerged as an extremely versatile platform for controlled drug release and targeted drug delivery, in which the route of administration is a key factor affecting therapeutic outcome. This strategy could be useful for delivering therapeutic agents to the central nervous system to treat neurological diseases [355,356], for promoting the penetration of drugs through ocular tissues, enhancing the therapeutic effect in diseases such as glaucoma and macular degeneration, nasal delivery of therapeutics to the brain through the olfactory nerve pathway, bypassing the BBB and delivering 5-fluorouracil as a topical treatment for ocular cancer [357,358,359]. While these approaches present significant therapeutic potential, research underscores the importance of a comprehensive evaluation of NP-induced irritation and toxicity in mucosal tissues [360,361,362].

The choice of PSNs for the administration route is based on various factors, including their physicochemical properties, the disease to be treated, and the desired release kinetics. For example, these can be functionalised for mucosal delivery, allowing mucoadhesion and escape from immune recognition associated with systemic delivery [363]. By modifying these features, PSNs can overcome biological barriers to achieve precise drug delivery, thereby improving the efficacy of drug treatment.

#### 2.4.6. Biodegradation and Clearance

**Figure 7:**
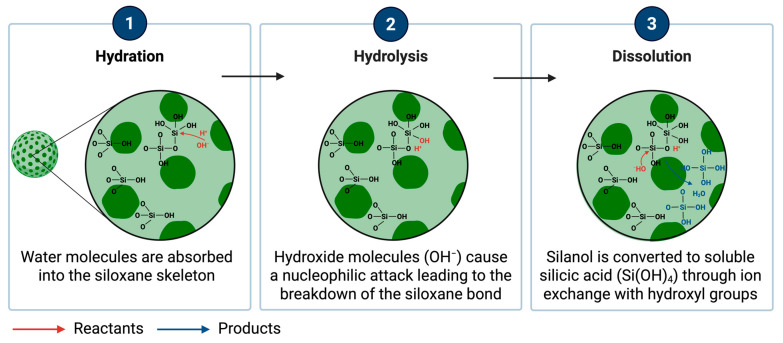
Biodegradation of PSNs (Created in BioRender. Patel, T. (2025) https://BioRender.com/q18ximj, reproduced from [5]. This figure demonstrates the steps in biodegradation. Step 1 shows hydration, step 2 shows hydrolysis, and step 3 shows dissolution. The red text represents the reactants involved in the breakdown reactions, whilst the blue text represents the products formed during degradation.

An obvious hindrance to PSNs serving as delivery vehicles is their poor biodegradation. PSNs could accumulate within nearby healthy tissues, potentially inducing toxic effects. The mechanism for biodegradation of PSNs is described as a three-step process. This occurs via a nucleophilic reaction between hydroxyl groups in aqueous media and non-bridged oxygen atoms on the PSN surface. The three steps of PSN degradation (Figure 7) are as follows: Step 1 outlines the hydration phase, in which the siloxane skeleton takes up water molecules. Step 2 is hydrolysis, where Si-O-Si bonds are cleaved to form silanol groups. Step 3 is dissolution, the conversion of silanol to soluble orthosilicic acid via ion exchange with hydroxy groups [364]. The resulting silicic acid is water-soluble and can be excreted through renal clearance; however, incomplete degradation can result in accumulation in the liver, spleen or lungs, contributing to chronic toxicity.

The rate and extent of PSN degradation depend strongly on physicochemical parameters and environmental factors such as pH and ionic strength. Smaller particles with thinner pore walls and hydrophilic surfaces typically degrade faster, while surface coatings or protein corona formation may hinder hydrolysis by shielding reactive sites. Acidic or enzymatic conditions, such as those in lysosomes, accelerate degradation, whereas neutral physiological conditions slow it down [36].

In addition, the reconstruction of an organic-inorganic structure could significantly enhance PSNs degradation. For example, co-condensation with organosilanes can introduce cleavable Si-C or Si-S bonds, making the framework amenable under physiological conditions. Similarly, doping with biodegradable elements such as calcium, zinc, or iron can weaken the Si-O-Si bonds and promote hydrolytic dissolution. The development of stimuli-responsive PSNs that degrade under acidic, redox, or enzyme-triggered environments could further enhance their safety profile [191]. Ultimately, understanding and optimizing PSN degradation is crucial to ensure therapeutic efficacy while maintaining biocompatibility and facilitating clinical translation.

**Figure 8:**
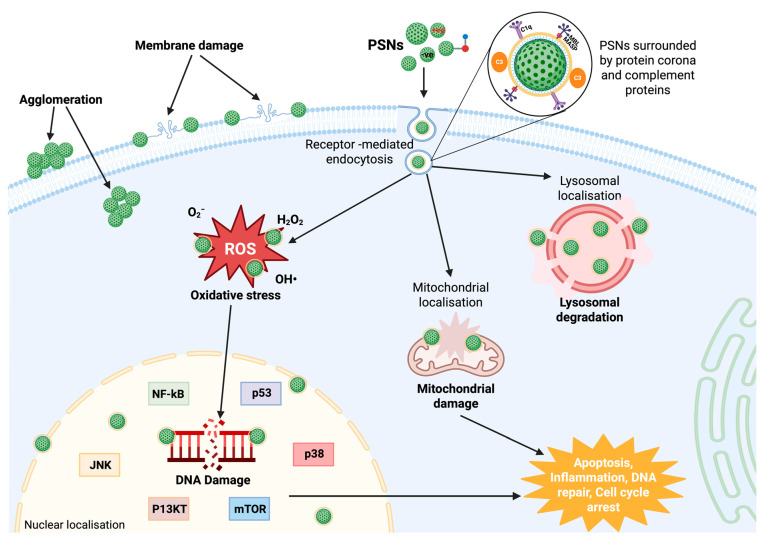
Overview of the nanotoxicity of PSNs (Created in BioRender. Patel, T. (2025) https://BioRender.com/4uz6rh2). This schematic diagram illustrates the cellular responses of PSNs, highlighting membrane damage, agglomeration, protein corona formation (yellow ring), activation of the complement system, cellular uptake, induction of oxidative stress leading to cytotoxic and genotoxic effects as well as possible cell signalling pathways activated by the toxicity of PSNs.

To visualize the toxicity endpoints and mechanisms discussed, Figure 8 outlines the possible sequence of events following PSN exposure, from surface interactions and internalization to intracellular responses such as oxidative stress, DNA damage, and activation of stress-related signalling cascades that lead to apoptosis, inflammation, DNA repair, and cell cycle arrest.

### 2.5. Evaluation of Clinical Translation

Evaluation of clinical translation is a critical component of SbD approaches, ensuring that PSNs can progress from preclinical research to clinical trials [28,365,366]. This stage involves assessing relevant animal studies, that reflect human physiology [9]. In vivo animal studies are critical for establishing the pharmacokinetic behaviour of PSNs in whole organisms. However, there is limited published data on PSNs assessed in larger animals; hence, more studies are needed to bridge the gap between rodent models and larger animals to enable the clinical translation of PSNs under the S.A.F.E. framework [5,367]. Also, various international bodies are working to ensure the safety of NPs and are moving toward establishing regulatory frameworks to address their potential risks. The Organisation for Economic Co-operation and Development (OECD) has developed guidelines for testing nanomaterials to standardize safety assessments and ensure the responsible development of nanotechnologies [368,369,370]. Similarly, the European Union’s Scientific Committee on Emerging and Newly Identified Health Risks provides scientific opinions on emerging health risks posed by new materials, including nanomaterials such as PSNs, especially in consumer products and medical applications [371]. Likewise, the United States Food and Drug Administration (FDA) issued NP-focused guidance for drug and medical device approval, with greater emphasis on physicochemical characterization, toxicity, and biodistribution [372,373]. Furthermore, the International Organization for Standardization (ISO) has developed standards such as ISO/TR 10993-22:2017, which provide protocols for assessing the biological effects of nanomaterials in medical devices [374]. These regulatory efforts highlight the need for interdisciplinary collaboration to address the safety concerns associated with PSNs, ensuring their safe and effective use in biomedical and industrial applications [375].

Additionally, considerations for manufacturing scalability and costs determine the practicality of translating laboratory-scale synthesis to industrial manufacturing, while maintaining material and performance consistency. Quality control measures are needed to avoid batch-to-batch variability and regulatory compliance [376]. Problems with reproducibility and size control continue to be an active area of research [377,378,379]. Finally, effective risk governance frameworks, including risk assessments, management, and communication throughout the life cycle, are vital to support informed decision-making and promote the clinical development of PSNs [380,381].

## 3. Summary

Many investigations have emphasized the importance of in-depth characterization of drug delivery-based NPs [75,382]. PSN characterization is essential for determining how it correlates with biological interactions, particularly in its role as a drug-delivery vehicle. In summary, the detailed characterization of PSNs is crucial for predicting and controlling their toxicity in humans. Data on particle size, pore size, functionalisation, surface charge, and crystallinity are key to understanding the utility of PSNs and enabling their effective and safe use.

Integrating findings on cellular uptake, protein corona dynamics, and immunogenic responses is essential for evaluating the biocompatibility and safety of PSNs. Cellular uptake efficiency determines the NP’s therapeutic potential [383], while the protein corona influences cellular interactions and immune responses [384]. Therefore, it is important to understand the interplay between these factors in PSNs, as this would dictate the overall biological outcome, including potential toxicity or therapeutic success [385]. By combining these findings, future studies will provide deeper insights into optimizing PSN design for safe and effective biomedical applications and the potential consequences of immune system activation. Understanding the principles of cytotoxicity assays and the various parameters that affect cytotoxicity underscores the importance of cytotoxicity testing. Surface functionalisation, exposure time, and the selection of diverse cell lines each play crucial roles in determining the safety and behaviour of PSNs within biological systems. Also, incorporating genotoxicity testing, oxidative stress assessments, and gene expression analysis into PSN toxicity studies is crucial for a thorough understanding of their biological impacts. These complementary approaches provide a robust evaluation of PSN interactions with cellular environments. They are essential for predicting potential health risks and advancing their application in biomedical fields while minimizing adverse effects. This multifaceted approach meets regulatory requirements and supports the development of safer, more effective PSNs for biomedical applications.

Finally, the S.A.F.E. PSNs framework (Table 4) provides a structured approach to developing and evaluating PSNs within SbD principles, ensuring their safety, efficacy and regulatory compliance. By integrating standardized characterization, toxicity assessment, relevant exposure models and clinical translation, this framework promotes responsible innovation while bridging the gap between laboratory research and real-world application. Ultimately, coordinated international regulatory effects and risk governance are key to supporting the safe and sustainable development of PSNs for clinical and industrial use. A summary (Table 4) highlighting current knowledge gaps and key research questions has been included, outlining areas where research remains limited and where future investigations should focus to advance PSN design and support safe clinical translation.

## Figures and Tables

**Table 1:** Porous and Mesoporous Silica Nanoparticle Applications in Various Clinical Trials.

Clinical Trial Study	Clinical Trial ID	Current Status	Study Type and Phase	Application	Purpose	Outcome
**Safety Evaluation of Porous Silica in Men**	NCT03667430 [9,10]	Completed in 2016	Interventional and Not applicable	Weight Loss	To evaluate the safety and tolerability of oral administration of PSNs in healthy men, primarily for applications in weight management and metabolic health.	Completed: Oral intake up to 9 g/day of porous silica as a food additive was safe and well-tolerated, with only mild gastrointestinal side effects and no major adverse events reported.
**Effect of Different NanoScaffolds on Pulp Regeneration in Non-Vital Immature Permanent Teeth**	NCT07121348 [11]	Active, not recruiting	Interventional and Not applicable	Regenerative Medicine	To evaluate the clinical and radiographic outcomes of regenerative endodontic procedures in immature non-vital permanent teeth using different nanoscaffold materials, including MSNs.	Ongoing: Outcome measures include radiographic apical closure 12 months post-procedure. No published results yet.
**Targeted Silica Nanoparticles for Real-Time Image-Guided Intraoperative Mapping of Nodal Metastases**	NCT02106598 [12,13]	Active, not recruiting	Interventional and Phase 1, Phase 2	Drug Delivery	To assess the feasibility and safety of using ultrasmall targeted core-shell silica nanoparticles for fluorescence-guided sentinel lymph node biopsy in patients with melanoma of the head and neck.	Ongoing: Nanoparticles enabled high-sensitivity lymph node mapping. No adverse events were observed. The approach was feasible and safe, offering improved intraoperative guidance.
**STAR Study Investigating Performance and Safety of the Medical Device SiPore15^TM^**	NCT03823027 [9,14,15]	Completed in 2019	Interventional and Not applicable	Diabetes	To evaluate the performance and safety of engineered MSN (SiPore15^TM^) in reducing blood glucose (HbA1c) in overweight or obese subjects with prediabetes or newly diagnosed type 2 diabetes.	Completed: SiPore15 ^TM^ significantly reduced HbA1c without affecting body weight, showing no serious adverse effects. Demonstrating its potential in controlling glycaemia.

**Table 2:** Comparison of the different cytotoxicity assays.

	Assays	MTT Assay	LDH Assay	Trypan Blue Exclusion Assay	Caspase Activity Assay	Annexin V/PI Staining Assay	Alamar Blue Assay	Cytokinesis Block Proliferation Index and Relative Population Doubling
Features								
**Type of Cytotoxicity Measured**		Metabolic Activity (Cell Viability)	Membrane Integrity (Cell Damage)	Membrane Integrity (Cell Damage)	Apoptosis (Programmed Cell Death)	Cell Death (Early Apoptosis, Late Apoptosis, Necrosis)	Metabolic Activity (Cell Viability)	Cytostasis (Reduction in growth rate)
**Test System**		Mammalian cells e.g. human lymphocytes and human hepatocytes	Mammalian cells e.g. human lymphocytes and human hepatocytes	Mammalian cells in suspension e.g. human lymphocytes and human hepatocytes	Mammalian cells e.g. human lymphocytes and human hepatocytes	Mammalian cells in suspension e.g. human lymphocytes and human hepatocytes	Mammalian cells e.g. human lymphocytes and human hepatocytes	Mammalian cells e.g. human lymphocytes and human hepatocytes
**Principle**		Yellow MTT tetrazolium salt is reduced to an insoluble purple formazan product by mitochondrial dehydrogenases in metabolically active cells.	LDH enzyme is released from damaged cells into the culture medium, which is measured as an indication of membrane integrity loss.	Living cells, which have an intact membrane, exclude the dye, whilst dead cells with damaged membranes take up the blue dye.	Measures the activation of caspase-3 and caspase-9, which are central drivers of apoptosis execution.	Annexin V labels early apoptosis (translocated phosphatidylserine). Propidium Iodide labels late apoptosis/necrosis (damaged membrane)	Blue resazurin is reduced to pink resorufin by metabolically active cells, producing a colourimetric or fluorescent signal.	To measure the average number of nuclear divisions a cell population has completed by scoring the frequency of cell with one, two or three nuclei after the addition of Cyto B to prevent cytokinesis.
**Advantages**		Highly sensitive and reproducible. Measures the metabolic activity of living cells. Widely used and well-established.	Simple and quick. Detects subtle background membrane damage.	Simple, inexpensive, and rapid. Suitable for high-throughput applications. Direct visualisation of viable and non-viable cells.	Directly measures apoptosis-specific pathways. Sensitive detection of early apoptotic events.	Differentiates between early apoptosis, late apoptosis, and necrosis. Quantitative and qualitative analysis via flow cytometry.	Non-toxic and allows continuous monitoring. Does not produce insoluble products. High sensitivity; ideal for high-throughput screening.	Can analyse cytotoxicity along with genotoxicity. Provides a more accurate measure of the average number of cells than simple counting.
**Disadvantages**		Insoluble formazan crystals require solubilisation. Metabolic activity may not always correlate with actual cell viability.	Cannot differentiate between necrosis and apoptosis.	Does not distinguish between apoptotic and necrotic cells. May overestimate dead cells due to transient membrane permeability.	Cannot detect necrosis or caspase-independent apoptosis. May miss late-stage cell death.	Requires precise flow cytometry gating. May generate false positives under non-apoptotic stress conditions. Transitional cell states may complicate interpretation (early and late apoptosis).	Metabolic activity may not directly equate to cell viability. Potential dye interactions with NPs; slower signal development.	Requires optimisation of concentration and exposure time of Cyto B. Labour intensive scoring. Only suitable for dividing cells.
**Relevance for PSNs/NPs**		Easy and quick method for identifying NP cytotoxicity with various doses of test agents at different time points.	Assess the membrane integrity damage induced by NPs.	Quick and high-volume screening of NP cytotoxicity based on membrane damage.	It can be used to investigate the apoptotic pathways triggered by NPs and can determine the underlying mechanism of cell death when combined with ROS or ER stress markers.	Distinguishes apoptosis from necrosis and can help identify which pathway is triggered.	An alternative to the MTT assay for assessing cell viability, especially if NP interference occurs.	Applicable with sequential addition of Cyto B to ensure no interference with NP endocytosis

**Table 3:** Comparison of the different genotoxicity assays.

	Assays	Cytokinesis Block Micronucleus (CBMN) Assay	Comet Assay	Chromosome Aberration Test	γ-H2AX Assay	Ames Test
Features						
**Type of Damage Detected**		Clastogenicity (chromosome breakage) and aneugenicity (chromosome loss)	DNA single-strand breaks, double-strand breaks and oxidative lesions	Structural chromosomal changes (breaks, deletions and rearrangements)	DNA double-strand breaks	Gene mutations (point mutations). Specifically base- pair substitutions and frameshift mutations.
**Test System**		Mammalian cells (e.g. human lymphocytes and human hepatocytes	Mammalian cells (e.g. human lymphocytes and human hepatocytes	Mammalian cells (e.g. human lymphocytes and human hepatocytes	Mammalian cells (e.g. human lymphocytes and human hepatocytes	Bacterial cells (e.g. Salmonella typhimurium and Escherichia coli)
**Principle**		Detects chromosome breakage or loss by scoring micronuclei in binucleated cells after cytokinesis is blocked.	Measures DNA strand breakage by electrophoretic migration of DNA fragments, forming a ‘comet tail’.	Identifies structural or numerical chromosome changes in cells arrested at metaphase after exposure to test agent.	Detects DNA double-strand breaks by quantifying phosphorylated histone H2AX foci.	Assesses mutagenicity of chemicals by measuring reversion mutations in bacterial strains.
**Advantages**		Simple and cost effective. High through-put analysis available to overcome manual scoring.	Single-cell analysis possible. Highly sensitive to low levels of DNA damage.	Structural and numerical aberrations are detected. Type of damage can be identified.	Allows early detection of DNA damage. Quantifiable using immunofluorescence or flow cytometry. Highly sensitive.	Quick and cost effective. Suitable for chemical mutagens.
**Disadvantages**		Requires actively dividing cells. Time-consuming and subjective quantification of micronuclei.	Cannot differentiate between clastogenic and aneugenic events	Labour intensive. Less sensitive to low level or transient DNA alterations	May detect non-relevant background phosphorylation. Does not detect all types of DNA damage.	Not suitable for nanoparticle assessment due to poor nanoparticle penetration into the bacterial cells. May produce false negatives. Cannot be done on its own; needs another assay alongside.
**Relevance for PSNs/NPs**		Measures DNA damage in daughter cells, which can help assess the long-term genomic instability from NP exposure	Useful for detecting early DNA damage by NPs as they can generate ROS or directly interact with DNA causing chromosomal damage	Detects chromosome changes caused by NPs, identifying carcinogenic and heritable effects of NP exposure	Can provide mechanistic insight into genotoxic stress pathways activated by NPs	Quick screening assay to identify mutagenicity alongside mammalian cell-based assays

**Table 4:** Research questions and current knowledge gaps.

Category	Research Question	Current Knowledge and Gaps
**Sample Preparation**	What is the guidance for sample preparation with advanced nanomaterials which do not dissolve? Can a universal dissolution protocol be established for inter-study comparison?	Studies often use different buffers and pH. A single standardised dissolution assay/protocol could allow reproducibility across research and maintain stability.
**Manufacturing**	Can scalable, reproducible synthesis methods be developed?	Microemulsion, sol-gel and template methods exist; however, there is batch-to-batch variability. A method is needed to maintain consistent pore size, structure and surface chemistry.
**Toxicity**	Can the silica matrix of PSNs be safely excreted or metabolised? How does its stability influence degradation pathways in biological environments?	PSNs undergo biodegradation primarily through excretion via the kidneys. However, the structural stability of the silica matrix, the rate and completeness of degradation that depends on physicochemical properties and long-term bioaccumulation, remains insufficiently understood.
	How do PSNs and their protein corona influence immunogenicity and potential immunotoxicity?	PSNs may activate the complement system or cytokine release depending on their physicochemical properties. However, comprehensive immunotoxicity studies of PSNs are limited. Standardised immune assays and long-term models are needed to clarify chronic immune effects and safety.
	What is the extent of biodistribution/accumulation of PSNs in major organs and tissues?	Short-term studies show accumulation in the liver, spleen and lungs with gradual clearance, but data on biodegradation rate and organ-specific accumulation after repeated dosing are limited. Comprehensive pharmacokinetic and chronic exposure studies are required.
	What are the long-term safety implications of PSNs in humans?	Preclinical studies show acute toxicity is low, but chronic toxicity has not been extensively studied. Standardised safety endpoints for PSNs must be established.
**Standardisation and Regulatory Challenges**	Are regulatory and standardisation frameworks sufficient for PSN translation? How can PSNs be integrated into regulatory frameworks for medical devices or drug formulation?	Regulatory guidance exists for some advanced nanomaterials, but not specifically for PSNs. Standardised characterisation, dissolution testing, toxicity endpoints and manufacturing methods need to be established to meet clinical trial requirements.

## Data Availability

No new data was created or analyzed in this manuscript.

## Supplementary Materials

The following supporting information can be downloaded at: https://www.mdpi.com/article/10.3390/nano15231766/s1. Section S1: Cytotoxicity assays and Section S2: Genotoxicity assays.

## Abbreviations

The following abbreviations are used in this manuscript:

α	Alpha
β	Beta
OH	Hydroxyl radical
16HBE14o	Human bronchial epithelial cells
3T3	Mouse fibroblast cells
A2780	Human ovarian cancer cells
A549	Human lung cancer cells
AgNPs	Silver nanoparticles
AKT	Protein kinase B
AMPK	Adenosine monophosphate-activated protein kinase
APC	Antigen presenting cells
Atg	Autophagy-related genes
ATM	Ataxia telangiectasia mutated
ATR	Ataxia telangiectasia and Rad3-related
AuNP	Gold nanoparticle
BAD	Bcl2-associated agonist of cell death
BAK	B-cell lymphoma 2 (BCL-2) antagonist
BAL	Human bronchioalveolar cells
BAX	BCL-2-associated X protein
BBB	Blood–brain barrier
Bcl-2	B-cell lymphoma 2
Bcl-XL	B-cell lymphoma 2-extra-large
BEAS-2B	Human bronchial epithelial cells
BSA	Bovine serum albumin
C3	Complement protein 3
C3b	Complement protein 3b
C5	Complement 5 protein
C6	Complement 6 protein
C7	Complement 7 protein
C8	Complement 8 protein
C9	Complement 9 protein
Caco-2	Human colon cancer cells
Calu-3	Human lung adenocarcinoma cells
CBMN	Cytokinesis block micronucleus
CDK	Cyclin-dependent kinase
CDK2	Cyclin-dependent kinase 2
DCFDA	2′,7′-dichlorofluorescein diacetate
DLS	Dynamic light scattering
DRAM1	DNA Damage-Regulated Autophagy Modulator 1
e.g.,	Exempli gratia, for example
EPR	Enhanced permeability and retention
ERK1	Extracellular-signal-regulating kinase 1
ERK2	Extracellular-signal-regulating kinase 2
et al.	Et alii
FDA	Food and Drug Administration
GIT	Gastrointestinal tract
GPx	Glutathione peroxidase
GRO-α	Growth-regulated oncogene alpha
H_2_O_2_	Hydrogen peroxide
HeLa	Human cervical cancer cells
HepG2	Human hepatocellular carcinoma cells
h	hours
HT-29	Human colorectal adenocarcinoma-derived cells
HUVEC	Human umbilical vein endothelial cells
IL-1β	Interleukin 1β
IL-6	Interleukin 6
IONP	Iron oxide nanoparticle
ISO	International Organisation for Standardisation
JNK	c-Jun-terminal kinase
MAC	Membrane attack complex
MAPK	Mitogen-activated protein kinase
MASP	Mannose-binding lectin associated serine proteases
MBL	Mannose binding lectin
MCF-7	Human breast cancer cells
MDA-MB 231	Human breast cancer cells
MDM2	Murine double minute 2
MOMP	Mitochondrial outer membrane permeabilisation
MPS	Mononuclear phagocyte system
MSN	Mesoporous silica nanoparticle
mTOR	Mammalian Target of Rapamycin
MTT	3- (4,5-dimethylthiazol-2-yl)-2,5-diphenyltetrazolium bromide
NF-kB	Nuclear factor kappa B
NINP	Niobium oxide nanoparticle
NiNP	Nickel nanoparticle
NLRP3	NOD-like receptor protein 3
NOXA	Phorbal-12-myristate-13-acetate-induced protein 1
NP	Nanoparticle
O_2_•^−^	Superoxide anion
OECD	Organisation for Economic Co-Operation and Development
p-NF-kB	Phosphorylated nuclear factor kappa B
P13K	Phosphatidylinositol-4,5-bisphosphate 3-kinase
p21	Cyclin-dependent kinase inhibitor 1A (CKDN1A)
p53	Tumour suppressor protein
PEG	Polyethylene glycol
PEI	Polyethylenimine
PDGF-AA	Platelet-derived growth factor-AA
PI	Propidium Iodide
PSN	Porous silica nanoparticle
PUMA	p53 upregulated modulator of apoptosis
ROS	Reactive oxygen species
SbD	Safe-by-Design
SDS-PAGE	Sodium dodecyl sulphate-polyacrylamide gel electrophoresis
Si	Silicon
SiNP	Silica nanoparticle
SiO_2_NP	Silicon dioxide nanoparticle
SOD	Superoxide dismutase
SWCNT	Single-walled carbon nanotube
THP-1	Human monocyte cells
TGF-1	Transforming growth factor beta 1
TiO_2_	Titanium dioxide
TK6	Human lymphocyte cells
TNF-α	Tumour necrotic factor-α
TP53	Tumour suppressor gene
TSC1	Hamartin
TSC2	Tuberin
ZnO	Zinc oxide
µg	Microgram
