# Supplementary material for: Nanotoxicity of Porous Silica Nanoparticles: Physicochemical Properties and Mechanistic Cellular Endpoints
Source: Nanomaterials (Basel). 2025 Nov 25;15(23):1766. doi: 10.3390/nano15231766 (PMC12693477; doi:10.3390/nano15231766)
Supplement: Supplementary file 1 [file nanomaterials-15-01766-s001.zip › nanomaterials-3959137-supplementary.pdf]

nanomaterials

MDPI

Review

Nanotoxicity of Porous Silica Nanoparticles: Physicochemical Properties and Mechanistic Cellular Endpoints

Trisha Patel [1], Callum Clipstone [1], Umakhanth Venkatraman Girija [1], Zeeshan Ahmad [2,3] and Neenu Singh [1,*]

[1] Leicester School of Allied Health Sciences, De Montfort University, Leicester, LE1 9BH, UK; trishapatel031@gmail.com (T.P.); p2601417@my365.dmu.ac.uk (C.C.); umakhanth.venkatramangirija@dmu.ac.uk (U.V.G.)
[2] Division of Pharmacy and Optometry, School of Health Sciences, University of Manchester, Manchester, M13 9PL, UK;
zeeshan.ahmad@manchester.ac.uk
[3] Leicester School of Pharmacy, De Montfort University, Leicester, LE1 9BH, UK
* Correspondence: neenu.singh@dmu.ac.uk

Supplementary Material

1. Cytotoxicity assays

1.1. MTT Assay

The MTT Assay (3-(4,5-dimethylthiazol-2-yl)-2,5-diphenyltetrazolium bromide) is among the most used assays to determine cell viability. The assay is based on the activity of mitochondrial dehydrogenases present in metabolically active cells. These enzymes act to reduce the yellow tetrazolium salt (MTT) to insoluble purple formazan crystals; the quantity of formazan formed is quantified by means of absorbance measurement, which correlates directly with the number of living cells [1]. Many studies have used this assay to identify NP cytotoxicity [2–5]. For example, the MTT assay was employed to assess cytotoxicity in triple-negative breast cancer (TNBC) cells following exposure to AuNPs. The study showed that negatively charged and positively charged AuNPs induced cell death in a dose-dependent manner [6]. Another study assessed the cytotoxicity of SiO_2 NPs in A549 lung cancer cells using the MTT assay. Results showed that cell viability decreased in a concentration-dependent manner, with viability ranging from 80% to 68% at 1-100 μg/mL concentrations after 24 hr treatment [7].

1.2. LDH Assay

The other common assay is the lactate dehydrogenase (LDH) assay. The release of the enzyme from injured cells into the extracellular environment is measured as an indication of membrane integrity loss [8,9]. There are multiple reasons why the LDH assay has advantages for measuring cell viability and cytotoxicity. One major strength is its consistency and reliability in detecting cellular damage, instilling confidence in researchers regarding their results. The assay is simple to assess, as LDH released to the culture medium correlates directly with the degree of cell death or damage. An additional benefit is the assay's speed; it provides a fast assessment of either cell viability or cytotoxicity within a short time frame. The LDH assay can also detect subtle background levels of cell membrane damage that may not be detectable using another method [10].

1.3. Trypan Blue Assay

Another simple technique, the trypan blue exclusion test, provides an advantage for high-throughput cytotoxicity screens, where estimating cell viability could be performed quickly with a haemocytometer or an automated cell counter. The trypan blue exclusion

test is a simple dye-based method used to determine cell viability based on membrane integrity [11]. In this assay, the dye is excluded from live cells, as their membranes are intact, but dead cells take up the dye, appearing blue when examined microscopically. The high-throughput trypan blue exclusion assay can be automated by mixing a cell suspension with the dye and using an automated cell counter or image cytometer to capture images where samples can be differentiated quickly between live and dead cells [12]. However, the method has limitations, such that it cannot discriminate between different types of cell death and does not differentiate between necrotic and apoptotic cells. Since both apoptotic and necrotic cells eventually lose membrane integrity and will be stained with the dye, the detection of trypan blue staining alone provides no insight into the mechanism of cell death [13]. In addition, rather harmless transient alterations in membrane permeability can occur, which are not necessarily indicative of cell death and result in the overestimation of non-viable cells.

1.4. Caspase Activity Assay

Caspase activity assays are used for assessing apoptosis. These specific caspases, such as caspase-3 and caspase-9, are the central drivers of apoptosis execution; therefore, this assay is based on their activation [14]. Caspase-9 is an initiator caspase which is activated through the mitochondrial apoptotic pathway. This triggers executioner caspases, mainly caspase-3, which cleaves cellular proteins, leading to DNA fragmentation and apoptotic bodies [15,16]. For instance, in Neuro-2a cells, it was demonstrated that SiNPs triggered apoptosis through the activation of caspase cascades (increased caspase-3 activity). The researchers found that this process was mediated through both ROS generation and endoplasmic reticulum stress [17]. Also, SiNPs exposure activated caspase-3 and caspase-9 in mouse alveolar macrophages. Activation of these caspases was partially dependent on mitochondrial dysfunction, as shown by mitochondrial depolarisation [11]. However, a limitation of this assay is that it only reports on caspase-dependent cell death and cannot detect other types of cell death, such as necrosis or caspase-independent apoptosis [18].

1.5. Annexin V/ Propidium Iodide (PI) Staining

Another assay based on flow cytometry is the annexin V/PI staining assay that differentiates between different cell death stages by specific cell markers. In normal healthy cells, phosphatidylserine is found on the cytoplasmic surface of the cell membrane. However, during apoptosis, annexin V labels phosphatidylserine that has translocated to the outer layer of the cell membrane, while PI enters cells only in late apoptosis or necrosis, labelling cells with compromised membrane integrity [19,20]. The annexin V assay was used to investigate the cytotoxicity of SiNPs on glioblastoma cells. They observed that SiNPs significantly induced apoptosis or necrosis in a dose-dependent manner. After 24 and 48 hr treatment, there was a significant increase in the proportion of apoptotic and necrotic cells when compared with the control groups [21]. While useful, this assay has limitations. Annexin V may bind to cells undergoing other stress responses, resulting in false positives, and accurate discrimination between early and late apoptosis is dependent on precise flow cytometric gating [19,20]. Cells can also be in a transitional state during the process between life and death, which can result in a discrepancy in determining the precise stages of cell death [22,23].

1.6. Alamar Blue Assay

Another common assay for measuring the viability of cells based on their metabolic activity is the alamar blue assay. It is based on the reduction of resazurin to resorufin by metabolically active cells, catalysed by mitochondrial and cytosolic NADPH dehydrogenases, producing a colorimetric or fluorescent signal that proportionally correlates to the

number of viable cells [24,25]. Different from the MTT assay, alamar blue does not produce insoluble formazan crystals, which may minimise the risk of interference by some NPs. However, before selecting an appropriate viability assay, it is essential to assess the potential interference of the NPs used [3, 24]. Although the alamar blue assay can be efficient for assessing cell viability in the presence of different NPs, the assay has its own limitations. Metabolic activity can differ from cell viability, and therefore, the assay can lead to misinterpretation, and some NPs can interact with the assay dye, reducing the accuracy. Moreover, longer incubation times may be necessary to generate a detectable signal, resulting in reduced overall throughput of the assay in high-volume screening applications [26].

1.7. Cytokinesis Block Proliferation Index and Relative Population Doubling

The Cytokinesis Block Proliferation Index (CBPI) and Relative Population Doubling (RPD) are other parameters which can be used to estimate the cytotoxicity of NPs, particularly with the Cytokinesis Block Micronucleus (CBMN) assay [27]. CBPI is a measure of cell proliferation that quantifies the distribution of mononucleate, binucleate and multinucleated cells within a treated population. It is calculated based on the number of these cell types, providing an indication of cell division status [28]. A reduction in CBPI suggests that the NPs may inhibit cell proliferation, indicating potential cytotoxic effects [27]. In contrast, the RPD quantifies the extent of cell population growth relative to an untreated control, offering a measure of how effectively cells continue to divide after NP exposure. It is calculated using the ratio of population doubling between treated and control cells, with a lower RPD value indicating impaired proliferation [28,29]. Together, CBPI and RPD can be useful indicators of how NPs influence cellular health, providing additional insights into NP-induced cytotoxicity beyond standard viability assays mentioned before.

2. Genotoxicity assays

2.1. Cytokinesis Block Micronucleus Assay

A wide range of assays are conducted to assess NP-induced genotoxicity by detecting DNA damage, chromosomal aberrations and mutagenicity [30]. The CBMN assay is an established genotoxicity test based on the presence of micronuclei in binucleated cells resulting from chromatin fragments or even whole chromosomes that are not incorporated into daughter nuclei during cell division. This method blocks cytokinesis, usually with cytochalasin B, allowing only single division cells to be scored, providing information on clastogenic (chromosome breakage) and aneugenic (chromosome loss) events [31,32]. Although the CBMN assay provides a good measure of chromosomal damage and exhibits a correlation with cancer risk, it has disadvantages. It necessitates actively dividing cells that may limit its applicability to non-proliferative cell types, and the quantification of micronuclei is a time-consuming and subjective process [33,34]. To overcome manual scoring, this assay has been adapted for high-throughput screening by using an automated fluorescence microscope and image analysis software which scores the micronuclei in binucleated cells [35,36].

2.2. Comet Assay

The comet assay or single-cell gel electrophoresis (SCGE) is another popular technique for detecting single and double-strand DNA breaks. In this assay, cells are embedded in agarose and then lysed. The lysate is subjected to electrophoresis under alkaline conditions, which permits fragmented DNA to migrate out of the nucleus and leads to comet tail formation. The length of the tail corresponds to the amount of DNA damage [37,38]. Many studies have proven that the comet assay can be used to assess DNA damage in nanomaterials [39–44]. For instance, it was observed that there was DNA damage

by AgNPs in lymphocytes using the comet assay with 25 μg/mL giving the most significant increase in tail DNA%, (~4 fold) [45]. Other studies with silver, gold and platinum NPs found a size and composition-specific DNA damage effect with the comet assay [46,47]. Similarly, data from another study demonstrated the genotoxicity of cationic lipopeptide NPs in mice; increased levels of DNA damage were exhibited in the kidney, lungs and liver [48].

2.3. γ-. H2AX Assay

Other commonly used genotoxicity assays include the γ-H2AX assay, which detects the phosphorylated form of the histone variant H2AX, which accumulates quickly at the sites of DNA double-strand breaks. In general, this assay is performed using either immunofluorescence or flow cytometry to quantify γ-H2AX foci as a marker for DNA damage [49,50]. Studies on nickel (Ni) NPs and nickel oxide (NiO) NPs found an increase in γ-H2AX foci in BEAS-2B human bronchial epithelial cells. The effect was stronger for NiONPs than for NiNPs, genotoxicity was correlated with elevated ROS generation [51]. This assay is mostly specific for double-strand breaks, and while other types of DNA breaks can be detected (e.g., breaks caused by the topoisomerases or misincorporation of DNA), the assay may not detect other types of damage; moreover, background levels of γ-H2AX from normal cellular activity may obscure the interpretation [52].

2.4. Ames Test

The Ames test is a selective bacterial mutagenicity assay which measures the ability of NPs to induce genetic mutations by assessing reverse mutation rates in designated strains of bacteria [53,54]. However, its usefulness may be hindered by its design for use with chemical mutagens rather than particulate materials. The differences in bacterial metabolism relative to human cellular processes may limit its suitability to predict human genotoxicity. Indeed, AgNPs showed no mutagenic effects with the Ames test in different bacterial strains. However, the micronucleus assay showed dose-dependent genotoxicity in TK6 cells [55]. Several other studies also used the Ames test to evaluate the mutagenic potential of various NPs. For instance, research on indium tin oxide NPs showed negative results in the Ames test across multiple bacterial strains, suggesting a lack of mutagenesis. However, in the same study, genotoxic responses in the micronucleus assay were seen, indicating that while the Ames test may not detect mutagenicity in NPs, other assays might reveal genotoxic potential [56]. Likewise, a study on the genotoxicity of AuNPs did not show evidence of mutagenic effects through the Ames test. Transmission electron microscopy showed that the AuNPs did not penetrate bacterial cells, potentially accounting for negative Ames test results. On the contrary, mammalian DNA damage was identified with the comet assay and micronucleus assay, which revealed the inadequacy of the Ames assay to evaluate NP mutagenicity [57].

These results indicate that, although the Ames test is a standard assay for detecting gene mutations, it may not be appropriate for NP genotoxicity assessment. This limitation is possibly due to the inability of NPs to penetrate the bacterial cell wall, resulting in false-negative results. Hence, it is suggested that mammalian-based assays such as the micronucleus or the comet assay, be included to investigate genotoxicity in NPs thoroughly.

2.5. Chromosome Aberration Test

Additionally, the chromosome aberration test is used to evaluate the structural integrity of chromosomes by detecting changes, such as breaks, deletions, or rearrangements in cells exposed to potential genotoxic substances [58]. Yazdimamaghani et al. [59] showed that SiNPs increased chromosomal aberrations in human peripheral blood lymphocytes, indicating that SiNPs could hinder DNA repair mechanisms. Although this

assay is critical for detecting structural chromosomal damage, it is often labour intensive, requires expert cytogenetic analysis, and may lack the sensitivity to identify low level or transient chromosomal alterations [60].

Genotoxicity induced by NPs has significant implications, especially because long-term health effects may arise. Consequently, persistent DNA damage may result in tumorigenesis, reproductive toxicity and hereditary mutations [61]. Valdiglesias et al. [52] reported increased mutagenesis in vivo following chronic exposure to TiO_2NPs, thereby posing a potential cancer risk. These results highlight the need for an in-depth genotoxic assessment of NPs before their biomedical translation.

The genotoxic effects of NPs vary depending on their physicochemical properties and exposure conditions. Surface modifications, including PEGylation and controlled functionalisation, have been shown to mitigate the genotoxic effects of smaller, positively charged, crystalline NPs, which induce DNA damage. The application of advanced high-throughput screening technologies and multi-omics approaches may shed new light on the genetic events induced by NPs and their repair processes. By optimising NP design and providing regulatory frameworks that prioritise nanotechnology, researchers can explore the therapeutic potential and safety in biomedical use.

Reference

1. Ghasemi, M.; Turnbull, T.; Sebastian, S.; Kempson, I. The MTT Assay: Utility, Limitations, Pitfalls, and Interpretation in Bulk and Single-Cell Analysis. *Int. J. Mol. Sci.* **2021**, *22*, 12827, https://doi.org/10.3390/ijms222312827.
2. Satyavani, K.; Gurudeeban, S.; Ramanathan, T.; Balasubramanian, T. Toxicity Study of Silver Nanoparticles Synthesized from Suaeda Monoica on Hep-2 Cell Line. *Avicenna J. Med Biotechnol.* **2025**, *17*, 157–157, https://doi.org/10.18502/ajmb.v17i2.18567.
3. Abbasi, F.; Hashemi, H.; Samaei, M.R.; SavarDashtaki, A.; Azhdarpoor, A.; Fallahi, M.J. The synergistic interference effect of silica nanoparticles concentration and the wavelength of ELISA on the colorimetric assay of cell toxicity. *Sci. Rep.* **2021**, *11*, 1–11, https://doi.org/10.1038/s41598-021-92419-1.
4. Vijayakumar, S.; Ganesan, S. *In Vitro* Cytotoxicity Assay on Gold Nanoparticles with Different Stabilizing Agents. *J. Nanomater.* **2012**, *2012*, https://doi.org/10.1155/2012/734398.
5. Almutary, A.; Sanderson, B.J.S. The MTT and Crystal Violet Assays: Potential Confounders in Nanoparticle Toxicity Testing. *Int. J. Toxicol.* **2016**, *35*, 454–462, https://doi.org/10.1177/1091581816648906.
6. Surapaneni, S.K.; Bashir, S.; Tikoo, K. Gold nanoparticles-induced cytotoxicity in triple negative breast cancer involves different epigenetic alterations depending upon the surface charge. *Sci. Rep.* **2018**, *8*, 1–12, https://doi.org/10.1038/s41598-018-30541-3.
7. Peivandi, Z.; Shirazi, F.H.; Teimourian, S.; Farnam, G.; Babaei, V.; Mehrparvar, N.; Koohsari, N.; Ashtarinezhad, A. Silica nanoparticles-induced cytotoxicity and genotoxicity in A549 cell lines. *Sci. Rep.* **2024**, *14*, 1–8, https://doi.org/10.1038/s41598-024-65333-5.
8. Gu, Q.; Cuevas, E.; Ali, S.F.; Paule, M.G.; Krauthamer, V.; Jones, Y.; Zhang, Y. An Alternative In Vitro Method for Examining Nanoparticle-Induced Cytotoxicity. *Int. J. Toxicol.* **2019**, *38*, 385–394, https://doi.org/10.1177/1091581819859267.
9. Han, X.; Gelein, R.; Corson, N.; Wade-Mercer, P.; Jiang, J.; Biswas, P.; Finkelstein, J.N.; Elder, A.; Oberdörster, G. Validation of an LDH assay for assessing nanoparticle toxicity. *Toxicology* **2011**, *287*, 99–104, https://doi.org/10.1016/j.tox.2011.06.011.
10. 1Cox, M.C.; Mendes, R.; Silva, F.; Mendes, T.F.; Zelaya-Lazo, A.; Halwachs, K.; Purkal, J.J.; Isidro, I.A.; Félix, A.; Boghaert, E.R.; et al. Application of LDH assay for therapeutic efficacy evaluation of ex vivo tumor models. *Sci. Rep.* **2021**, *11*, 1–14, https://doi.org/10.1038/s41598-021-97894-0.
11. Thibodeau, M.; Giardina, C.; Hubbard, A.K. Silica-Induced Caspase Activation in Mouse Alveolar Macrophages Is Dependent upon Mitochondrial Integrity and Aspartic Proteolysis. *Toxicol. Sci.* **2003**, *76*, 91–101, https://doi.org/10.1093/toxsci/kfg178.
12. Bell, J.; Huang, Y.; Qazi, H.; Kuksin, D.; Qiu, J.; Lin, B.; Chan, L.L.-Y. Characterization of a novel high-throughput, high-speed and high-precision plate-based image cytometric cell counting method. *Cell Gene Ther. Insights* **2021**, *7*, 427–447, https://doi.org/10.18609/cgti.2021.070.
13. Strober, W. Trypan Blue Exclusion Test of Cell Viability. *Curr. Protoc. Immunol.* **2015**, *111*, A3.B.1–A3.B.3, https://doi.org/10.1002/0471142735.ima03bs111.
14. Nichani, K.; Li, J.; Suzuki, M.; Houston, J.P. Evaluation of Caspase-3 Activity During Apoptosis with Fluorescence Lifetime-Based Cytometry Measurements and Phasor Analyses. *Cytom. Part A* **2020**, *97*, 1265–1275, https://doi.org/10.1002/cyto.a.24207.

15. Chen, B.; Kiely, J.; Williams, I.; Luxton, R. A non-faradaic impedimetric biosensor for monitoring of caspase 9 in mammalian cell culture. *Bioelectrochemistry* **2023**, *153*, 108456, https://doi.org/10.1016/j.bioelechem.2023.108456.
16. Brentnall, M.; Rodriguez-Menocal, L.; De Guevara, R.L.; Cepero, E.; Boise, L.H. Caspase-9, caspase-3 and caspase-7 have distinct roles during intrinsic apoptosis. *BMC Cell Biol.* **2013**, *14*, 32, https://doi.org/10.1186/1471-2121-14-32.
17. Lee, K.-I.; Lin, J.-W.; Su, C.-C.; Fang, K.-M.; Yang, C.-Y.; Kuo, C.-Y.; Wu, C.-C.; Wu, C.-T.; Chen, Y.-W. Silica nanoparticles induce caspase-dependent apoptosis through reactive oxygen species-activated endoplasmic reticulum stress pathway in neuronal cells. *Toxicol. Vitr.* **2020**, *63*, 104739, https://doi.org/10.1016/j.tiv.2019.104739.
18. Lekshmi, A.; Varadarajan, S.N.; Lupitha, S.S.; Indira, D.; Mathew, K.A.; Nair, A.C.; Nair, M.; Prasad, T.; Sekar, H.; Gopalakrishnan, A.K.; et al. A quantitative real-time approach for discriminating apoptosis and necrosis. *Cell Death Discov.* **2017**, *3*, 16101, https://doi.org/10.1038/cddiscovery.2016.101.
19. Mattes, M.J. Apoptosis assays with lymphoma cell lines: problems and pitfalls. *Br. J. Cancer* **2007**, *96*, 928–936, https://doi.org/10.1038/sj.bjc.6603663.
20. Pischel, D.; Buchbinder, J.H.; Sundmacher, K.; Lavrik, I.N.; Flassig, R.J. A guide to automated apoptosis detection: How to make sense of imaging flow cytometry data. *PLOS ONE* **2018**, *13*, e0197208, https://doi.org/10.1371/journal.pone.0197208.
21. Krętowski, R.; Kusaczuk, M.; Naumowicz, M.; Cechowska-Pasko, M. The Pro-Apoptotic Effect of Silica Nanoparticles Depends on Their Size and Dose, as Well as the Type of Glioblastoma Cells. *Int. J. Mol. Sci.* **2021**, *22*, 3564, https://doi.org/10.3390/ijms22073564.
22. Elmore, S. Apoptosis: A review of programmed cell death. *Toxicol. Pathol.* **2007**, *35*, 495–516. doi:10.1080/01926230701320337.
23. Galluzzi, L.; Vitale, I.; Aaronson, S.A.; Abrams, J.M.; Adam, D.; Agostinis, P.; Alnemri, E.S.; Altucci, L.; Amelio, I.; Andrews, D.W.; et al. Molecular mechanisms of cell death: Recommendations of the Nomenclature Committee on Cell Death 2018. *Cell Death Differ.* **2018**, *25*, 486–541, doi:10.1038/s41418-017-0012-4.
24. Dinh, M.N.; Hitomi, M.; Al-Turaihi, Z.A.; Scott, J.G. Alamar Blue assay optimization to minimize drug interference and inter assay viability. *MethodsX* **2024**, *13*, 103024, https://doi.org/10.1016/j.mex.2024.103024.
25. Osmani, Z.; Islam, M.A.; Wang, F.; Meira, S.R.; Kulka, M. Optimization of a rapid, sensitive, and high throughput molecular sensor to measure canola protoplast respiratory metabolism as a means of screening nanomaterial cytotoxicity. *Plant Methods* **2024**, *20*, 1–14, https://doi.org/10.1186/s13007-024-01289-x.
26. Longhin, E.M.; El Yamani, N.; Rundén-Pran, E.; Dusinska, M. The alamar blue assay in the context of safety testing of nanomaterials. *Front. Toxicol.* **2022**, *4*, 981701, https://doi.org/10.3389/ftox.2022.981701.
27. Ruiz-Ruiz, B.; Arellano-García, M.E.; Radilla-Chávez, P.; Salas-Vargas, D.S.; Toledano-Magaña, Y.; Casillas-Figueroa, F.; Luna Vazquez-Gomez, R.; Pestryakov, A.; García-Ramos, J.C.; Bogdanchikova, N. Cytokinesis-Block Micronucleus Assay Using Human Lymphocytes as a Sensitive Tool for Cytotoxicity/Genotoxicity Evaluation of AgNPs. *ACS Omega* **2020**, *5*, 12005–12015, doi:10.1021/acsomega.0c00149.
28. OECD OECD Test Guideline No. 487 In Vitro Mammalian Cell Micronucleus Test Available online: http://www.oecd.org/termsandconditions/.
29. Lorge, E.; Hayashi, M.; Albertini, S.; Kirkland, D. Comparison of different methods for an accurate assessment of cytotoxicity in the in vitro micronucleus test. *Mutat. Res. Toxicol. Environ. Mutagen.* **2008**, *655*, 1–3, https://doi.org/10.1016/j.mrgentox.2008.06.003.
30. Fontaine, M.; Bartolami, E.; Prono, M.; Béal, D.; Blosi, M.; Costa, A.L.; Ravagli, C.; Baldi, G.; Sprio, S.; Tampieri, A.; et al. Nanomaterial genotoxicity evaluation using the high-throughput p53-binding protein 1 (53BP1) assay. *PLOS ONE* **2023**, *18*, e0288737, https://doi.org/10.1371/journal.pone.0288737.
31. Vallabani, N.V.S.; Karlsson, H.L. Primary and Secondary Genotoxicity of Nanoparticles: Establishing a Co-Culture Protocol for Assessing Micronucleus Using Flow Cytometry. *Front. Toxicol.* **2022**, *4*, 845987, https://doi.org/10.3389/ftox.2022.845987.
32. Sioen, S.; Cloet, K.; Vral, A.; Baeyens, A. The Cytokinesis-Block Micronucleus Assay on Human Isolated Fresh and Cryopreserved Peripheral Blood Mononuclear Cells. *J. Pers. Med.* **2020**, *10*, 125, https://doi.org/10.3390/jpm10030125.
33. Bertucci, A.; Wilkins, R.C.; Lachapelle, S.; Turner, H.C.; Brenner, D.J.; Garty, G. Comparison of Isolated Lymphocyte and Whole Blood-Based CBMN Assays for Radiation Triage. *Cytogenet. Genome Res.* **2023**, *163*, 110–120, https://doi.org/10.1159/000533488.
34. Shen, X.; Chen, Y.; Li, C.; Yang, F.; Wen, Z.; Zheng, J.; Zhou, Z. Rapid and automatic detection of micronuclei in binucleated lymphocytes image. *Sci. Rep.* **2022**, *12*, 1–14, https://doi.org/10.1038/s41598-022-07936-4.
35. Grzesiakowska-Dul, A.; Kasprowicz, M.J.; Otwinowska-Mindur, A.; Baran, P.; Kuchta-Gładysz, M. Cytokinesis-Blocking Micronucleus Assay for Assessing Nuclear Chromatin Integrity Abnormalities in Dog's Somatic Cells After Exposure to HVAD-Produced Silver Nanoparticles. *Int. J. Mol. Sci.* **2024**, *25*, 12691, https://doi.org/10.3390/ijms252312691.

36. Capaccio, C.; Perrier, J.R.; Cunha, L.; Mahnke, R.C.; Lörch, T.; Porter, M.; Smith, C.L.; Damer, K.; Bourland, J.D.; Frizzell, B.; et al. CytoRADx: A High-Throughput, Standardized Biodosimetry Diagnostic System Based on the Cytokinesis-Block Micronucleus Assay. *Radiat. Res.* **2021**, *196*, 523–534, https://doi.org/10.1667/rade-20-00030.1.
37. McKelvey-Martin, V.; Green, M.; Schmezer, P.; Pool-Zobel, B.; De Méo, M.; Collins, A. The single cell gel electrophoresis assay (comet assay): A European review. *Mutat. Res. Mol. Mech. Mutagen.* **1993**, *288*, 47–63, https://doi.org/10.1016/0027-5107(93)90207-v.
38. Olive, P.L.; Banáth, J.P. The comet assay: a method to measure DNA damage in individual cells. *Nat. Protoc.* **2006**, *1*, 23–29, https://doi.org/10.1038/nprot.2006.5.
39. Güneş, M.; Yalçın, B.; Ali, M.M.; Ciğerci, İ.H.; Kaya, B. Genotoxic assessment of cerium and magnesium nanoparticles and their ionic forms in *Eisenia hortensis* coelomocytes by alkaline comet assay. *Microsc. Res. Tech.* **2022**, *85*, 3095–3103, https://doi.org/10.1002/jemt.24168.
40. Ahamed, M.; Alhadlaq, H.A.; Alam, J.; Khan, M.A.M.; Ali, D.; Alarafi, S. Iron Oxide Nanoparticle-induced Oxidative Stress and Genotoxicity in Human Skin Epithelial and Lung Epithelial Cell Lines. *Curr. Pharm. Des.* **2013**, *19*, 6681–6690, https://doi.org/10.2174/1381612811319370011.
41. Pfuhler, S.; Downs, T.R.; Allemang, A.J.; Shan, Y.; Crosby, M.E. Weak silica nanomaterial-induced genotoxicity can be explained by indirect DNA damage as shown by the OGG1-modified comet assay and genomic analysis. *Mutagenesis* **2016**, *32*, 5–12, https://doi.org/10.1093/mutage/gew064.
42. Vandghanooni, S.; Eskandani, M. Comet Assay: A Method to Evaluate Genotoxicity of Nano-Drug Delivery System. **2011**, *1*, 87–97, https://doi.org/10.5681/bi.2011.012.
43. Barnes, C.A.; Elsaesser, A.; Arkusz, J.; Smok, A.; Palus, J.; Leśniak, A.; Salvati, A.; Hanrahan, J.P.; de Jong, W.H.; Dziubałtowska, E.; et al. Reproducible Comet Assay of Amorphous Silica Nanoparticles Detects No Genotoxicity. *Nano Lett.* **2008**, *8*, 3069–3074, https://doi.org/10.1021/nl801661w.
44. I Salama, M.; A El Morsi, D.; Shabka, O.; Emam, N.M. Assessment of genotoxicity of silver nanoparticles on lymphocyte cells of albino rats using comet assay. *Toxicol. Res.* **2021**, *10*, 1085–1091, https://doi.org/10.1093/toxres/tfab092.
45. Ghosh, M.; J, M.; Sinha, S.; Chakraborty, A.; Mallick, S.K.; Bandyopadhyay, M.; Mukherjee, A. In vitro and in vivo genotoxicity of silver nanoparticles. *Mutat. Res. Toxicol. Environ. Mutagen.* **2012**, *749*, 60–69, https://doi.org/10.1016/j.mrgentox.2012.08.007.
46. Kohl, Y.; Rundén-Pran, E.; Mariussen, E.; Hesler, M.; El Yamani, N.; Longhin, E.M.; Dusinska, M. Genotoxicity of Nanomaterials: Advanced In Vitro Models and High Throughput Methods for Human Hazard Assessment—A Review. *Nanomaterials* **2020**, *10*, 1911, https://doi.org/10.3390/nano10101911.
47. Lebedová, J.; Hedberg, Y.S.; Odnevall Wallinder, I.; Karlsson, H.L. Size-dependent genotoxicity of silver, gold and platinum nanoparticles studied using the mini-gel comet assay and micronucleus scoring with flow cytometry. *Mutagenesis* **2017**, *33*, 77–85, https://doi.org/10.1093/mutage/gex027.
48. Zhanataev, A.K.; Anisina, E.A.; Kulakova, A.V.; Shilovskiy, I.P.; Lisitsyn, A.A.; Koloskova, O.O.; Khaitov, M.R.; Durnev, A.D. Genotoxicity of cationic lipopeptide nanoparticles. *Toxicol. Lett.* **2020**, *328*, 1–6, https://doi.org/10.1016/j.toxlet.2020.04.011.
49. Heylmann, D.; Kaina, B. The γH2AX DNA damage assay from a drop of blood. *Sci. Rep.* **2016**, *6*, 22682, https://doi.org/10.1038/srep22682.
50. Wu, J.; Clingen, P.H.; Spanswick, V.J.; Mellinas-Gomez, M.; Meyer, T.; Puzanov, I.; Jodrell, D.; Hochhauser, D.; Hartley, J.A. γ-H2AX Foci Formation as a Pharmacodynamic Marker of DNA Damage Produced by DNA Cross-Linking Agents: Results from 2 Phase I Clinical Trials of SJG-136 (SG2000). *Clin. Cancer Res.* **2013**, *19*, 721–730, https://doi.org/10.1158/1078-0432.ccr-12-2529.
51. Åkerlund, E.; Cappellini, F.; Di Bucchianico, S.; Islam, S.; Skoglund, S.; Derr, R.; Wallinder, I.O.; Hendriks, G.; Karlsson, H.L. Genotoxic and mutagenic properties of Ni and NiO nanoparticles investigated by comet assay, γ-H2AX staining, Hprt mutation assay and ToxTracker reporter cell lines. *Environ. Mol. Mutagen.* **2017**, *59*, 211–222, https://doi.org/10.1002/em.22163.
52. Valdiglesias, V.; Giunta, S.; Fenech, M.; Neri, M.; Bonassi, S. γH2AX as a marker of DNA double strand breaks and genomic instability in human population studies. *Mutat. Res. Mol. Mech. Mutagen.* **2013**, *753*, 24–40, https://doi.org/10.1016/j.mrrev.2013.02.001.
53. Elespuru, R.; Pfuhler, S.; Aardema, M.J.; Chen, T.; Doak, S.H.; Doherty, A.; Farabaugh, C.S.; Kenny, J.; Manjanatha, M.; Mahadevan, B.; et al. Genotoxicity Assessment of Nanomaterials: Recommendations on Best Practices, Assays, and Methods. *Toxicol. Sci.* **2018**, *164*, 391–416, https://doi.org/10.1093/toxsci/kfy100.
54. Kim, H.R.; Park, Y.J.; Shin, D.Y.; Oh, S.M.; Chung, K.H. Appropriate In Vitro Methods for Genotoxicity Testing of Silver Nanoparticles. *Environ. Heal. Toxicol.* **2013**, *28*, e2013003, https://doi.org/10.5620/eht.2013.28.e2013003.

55. Li, Y.; Chen, D.H.; Yan, J.; Chen, Y.; Mittelstaedt, R.A.; Zhang, Y.; Biris, A.S.; Heflich, R.H.; Chen, T. Genotoxicity of silver nanoparticles evaluated using the Ames test and in vitro micronucleus assay. *Mutat. Res. Toxicol. Environ. Mutagen.* **2012**, *745*, 4–10, https://doi.org/10.1016/j.mrgentox.2011.11.010.
56. Akyıl, D.; Eren, Y.; Konuk, M.; Tepekozcan, A.; Sağlam, E. Determination of mutagenicity and genotoxicity of indium tin oxide nanoparticles using the Ames test and micronucleus assay. *Toxicol. Ind. Heal.* **2016**, *32*, 1720–1728, https://doi.org/10.1177/0748233715579804.
57. George, J.M.; Magogotya, M.; Vetten, M.A.; Buys, A.V.; Gulumian, M. An Investigation of the Genotoxicity and Interference of Gold Nanoparticles in Commonly Used*In Vitro*Mutagenicity and Genotoxicity Assays. *Toxicol. Sci.* **2017**, *156*, 149–166, https://doi.org/10.1093/toxsci/kfw247.
58. Paniagua, I.; Jacobs, J. Quantification of Chromosomal Aberrations in Mammalian Cells. *Bio-Protocol* **2023**, *13*, e4739, https://doi.org/10.21769/bioprotoc.4739.
59. Yazdimamaghani, M.; Moos, P.J.; Dobrovolskaia, M.A.; Ghandehari, H. Genotoxicity of amorphous silica nanoparticles: Status and prospects. *Nanomedicine: Nanotechnology, Biol. Med.* **2019**, *16*, 106–125, https://doi.org/10.1016/j.nano.2018.11.013.
60. Ahluwalia, K.K.; Thakur, K.; Ahluwalia, A.S.; Hashem, A.; Avila-Quezada, G.D.; Abd_Allah, E.F.; Thakur, N. Assessment of Genotoxicity of Zinc Oxide Nanoparticles Using Mosquito as Test Model. *Toxics* **2023**, *11*, 887, https://doi.org/10.3390/toxics11110887.
61. Xuan, L.; Ju, Z.; Skonieczna, M.; Zhou, P.; Huang, R. Nanoparticles-induced potential toxicity on human health: Applications, toxicity mechanisms, and evaluation models. *Medcomm* **2023**, *4*, e327, https://doi.org/10.1002/mco2.327.
